# Early stages of insulin fibrillogenesis examined with ion mobility mass spectrometry and molecular modelling†

**DOI:** 10.1039/c5an01253h

**Published:** 2015-09-09

**Authors:** Harriet Cole, Massimiliano Porrini, Ryan Morris, Tom Smith, Jason Kalapothakis, Stefan Weidt, C. Logan Mackay, Cait E. MacPhee, Perdita E. Barran

**Affiliations:** a EastChem School of Chemistry Joseph Black Building The King's Buildings West Mains Rd Edinburgh EH9 3JJ UK; b SUPA, School of Physics and Astronomy James Clark Maxwell Building The King's Buildings West Mains Rd Edinburgh EH9 3JZ UK; c Michael Barber Centre for Collaborative Mass Spectrometry, School of Chemistry, Manchester Institute of Mass Spectrometry, The University of Manchester Manchester M1 7DN UK perdita.barran@manchester.ac.uk +44(0) 161 275 0256

## Abstract

A prevalent type of protein misfolding causes the formation of β-sheet-rich structures known as amyloid fibrils. Research into the mechanisms of fibril formation has implications for both disease prevention and nanoscale templating technologies. This investigation into the aggregation of insulin utilises ion mobility mass spectrometry coupled with molecular modelling to identify and characterise oligomers formed during the ‘lag’ phase that precedes fibril growth. High resolution mass spectrometry and collision induced dissociation is used to unequivocally assign species as *m*/*z* coincident multimers or confomers, providing a robust analytical approach that supports the use of molecular dynamics to atomistically resolve the observed oligomers. We show that insulin oligomerises to form species I_*n*_ where 2 ≤ *n* ≤ 12 and within this set of oligomers we delineate over 60 distinct conformations, the most dominant of which are compact species. Modelling trained with experimental data suggests that the dominant compact dimers are enriched in β-sheet secondary structure and dominated by hydrophobic interactions, and provides a linear relationship between *R*_g_ and collision cross section. This approach provides detailed insight to the early stages of assembly of this much studied amyloidogenic protein, and can be used to inform models of nucleation and growth.

## Abstract 2

Mass spectrometry shows insulin oligomers [I]_n_ where n ranges from 1-12, and ion mobility analysis reveals ∼60 structurally distinct species across this oligomer distribution. Experimental data trains MD simulations to characterize a persistent prefibrillar protein oligomer that is a dimer enriched in β sheets.

## Graphical abstract

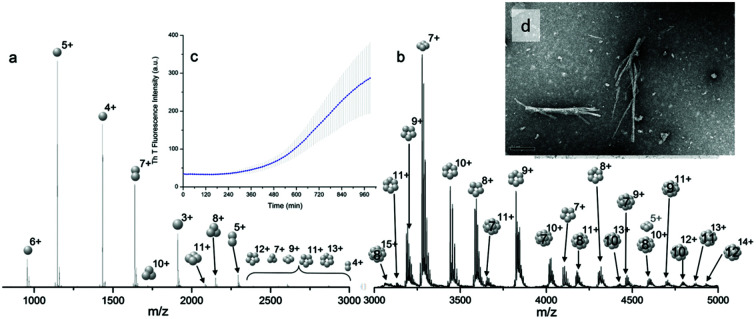

## Introduction

The formation and growth of fibrillar aggregates follows sigmoidal kinetics, comprising an initial lag phase followed by a period of accelerated growth to saturation. Such behaviour has been ascribed to a nucleation and growth process similar to that observed in crystallisation,^1^ however recent investigations by Knowles *et al.*^2^ suggest that nucleation may in fact be very rapid, and that the characteristic sigmoidal growth curve is a result of secondary processes, such as fibril breaking. During the early stages of self-assembly (the apparent “lag” phase) this model predicts that a wide variety of different oligomeric species will be present, all potentially on-pathway to self-assembly. What it cannot divulge is the conformation(s) of any given oligomeric species, and yet such information is critical not only to the development of a comprehensive model for assembly, but also to assist the development of small molecule inhibitors of aggregation. Here we use mass spectrometry (MS) and molecular dynamics (MD) to report with molecular detail on the oligomeric species present before the onset of fibril growth.

Insulin has been the focus of a large number of studies of the self-assembly of proteins into fibrillar aggregates.^3^ It is a predominantly α-helical protein in its native state, and is stored as a hexamer at neutral pH in the β cells of the pancreas. Rapid fibrillogenesis of insulin occurs *in vitro* at low pH and a temperature of 60 °C,^4^ both of which favour monomerisation and partial unfolding of the protein. An insulin monomer consists of two chains, chain A (21 amino acids) and chain B (30 amino acids) which are cross-linked by two disulphide bridges, with an additional intra-chain disulphide bond present in chain A.^5^

In the current study we identify the oligomers present in the lag phase by mass spectrometry (MS). MS is the only technique that can separate the species present in the lag phase, identifying the many transient oligomeric orders according to their mass to charge (*m*/*z*) ratio as well as revealing their relative abundance. Previous MS investigations under native-like (non fibril-forming) conditions have detected insulin oligomers up to hexamer^6^ and under aggregating conditions Nettleton *et al.* observed oligomers up to dodecamer.^3^ Ion mobility mass spectrometry (IM-MS) enhances this picture by probing the conformational landscape of the aggregating system. IM-MS separates species that are coincident in *m*/*z* space according to their differing mobilities, reflecting the size, shape and charge of the ions. The use of IM-MS to study the early oligomers formed in amyloidal systems has been pioneered by Bowers and co-workers.^7^ For example, Bleiholder *et al.* have used this approach to study structural transitions in small (∼6 amino acid), potentially amyloidogenic peptides.^8^ It has recently been applied to study potential fibrillation inhibitors,^9^ and to examine the flexibility of alpha synuclein,^10^ and structural rearrangements in apo-C-II.^10*a*^

IM-MS provides an arrival time distribution for any observed ion; this distribution may be multimodal, resolving multiple species that have the same *m*/*z*. These can be assigned as being either higher order aggregates of the general form *nm*/*nz* or conformers of the same *m*/*z* species, or indeed a mixture of both. So while the partial orthogonality of IM separation provides more detail that MS alone, correct assignment of multiple species is not trivial and is essential to the correct interpretation of the data. Resolved features in arrival time distributions can be converted to collision cross section data, which in turn can be used to gain insights to assembly (the route taken by Bleiholder *et al.*^8^) and also to couple with structures from MD simulations to provide atomistically resolved detail on plausible candidates for the observed conformers.

Both of these IM-MS enabled approaches are exciting new tools to improve our understanding of protein and peptide self-assembly, and to assist efforts in targeting particular toxic oligomers for drug discovery, but they rely critically on correct initial assignment of the value of *m* and *z* of the resolved species. High resolution mass spectrometry allows charge state assignment by providing isotopically resolved spectra, however many of the home built IM-MS devices, while possessing very high IM resolution compared with commercial IM-MS instruments, do not have high *m*/*z* resolution. Additional analysis is then needed to confirm identification, especially when this is being used to support growth mechanisms or to train MD simulations. This can be achieved by high resolution mass spectrometry measurements and/or with the use of collision induced dissociation to dissect the oligomers. In this study we use both of these methods to support our assignment of insulin oligomers with masses up to 34.5 kDa (hexamers), proving that such an approach is possible even for sizeable prefibrillar aggregates.

Our careful approach to assignment of the stable entities observed in IM-MS arrival time distributions allows us to gain unprecedented insight into the self assembly of insulin. We observe significantly populated conformational families of oligomers. The oligomeric order is confirmed with high resolution Fourier Transform Ion Cyclotron Resonance (FT-ICR) MS which unequivocally assigns *m*/*z* coincident species. We use these experimental data to train MD simulations, providing atomistic detail for the dominant dimeric species. The stability of several representative dimers is evaluated *via* binding energy calculations. Identifying the interaction between monomers is vital as dimer formation must be the first step in any self assembly pathway; furthermore the interface between interacting monomers may be the target site for small molecule drugs that could potentially prevent amyloid formation. For all oligomeric species observed, the most significantly populated conformation is the most compact form, and MD indicates that these may be enriched in β sheet.

## Experimental section

### Materials

Bovine insulin, CAS Number 11070-73-8 I5500, was obtained as a lyophilized powder from Sigma Aldrich and dissolved in water adjusted to pH2 with formic acid, and with HCl to pH 1.6. The zinc content of the powder was approximately 0.5% w/w. The samples were immediately flash frozen in liquid nitrogen and stored in a freezwer at −28 °C. The samples were left at room temperature for up to 24 hours prior to use, no change in the distribution of oligomers in the mass spectrometry data nor in the arrival time distributions were observed at a function of incubation time.

### Mass spectrometry

Tuning parameters on all instruments were optimised to facilitate the greatest preservation of oligomeric species. These parameters included cone voltage (Fig. S1, and S2†) concentration (Fig. S3 and S4†) collision energies, injection energies (for IM-MS, Fig. S5†) and source pressure. We also considered the effect of HCl to acidify the solution (Fig. S6†).

### Ion mobility mass spectrometry

Ion mobility mass spectrometry measurements were performed using an in-house modified QToF 1 (Micromass UK Ltd).^11^ Adaptations have enabled it to make temperature dependent collision cross section measurements, with the inclusion of a 5.1 cm long copper drift cell (filled with helium gas at a pressure of ∼3.5 Torr) and supplementary ion optics situated post source optics and before the quadrupole analyser. Ions are pulsed into the cell and drift through under the influence of a weak electric field, hindered by the intervening buffer gas molecules. Thus the time taken for ions to traverse the cell, in conjunction with the strength of the electric field applied across the drift cell, is a reflection of their associated charge and their mobility (*K*) which is inversely related to their rotationally averaged CCS (*Ω*).^12^ The ions then pass through the quadrupole analyser and the time of flight tube and are finally detected by microchannel plates. The interval between the time the ions are pulsed into the drift cell to the time they are detected is therefore a combination of the time the ions spend in the drift cell (drift time) and that outside it (dead time). This is known as the arrival time distribution (ATD), and can be deconvoluted into ATDs for each individual ion. For a given *m*/*z* the drift time will vary depending on its CCS but the dead time will be invariant. The ions ATD distributions are measured to 6 different values of the electric field applied to the drift cell (drift voltage). If the arrival times are plotted against *P*/*V* the intercept will be the dead time and the gradient 1/*K*. After normalising for the experimental temperature and pressure inside the drift cell *K* can be converted into reduced mobility *K*_0_ and this to ascertain a value for *Ω* using the equation below.1
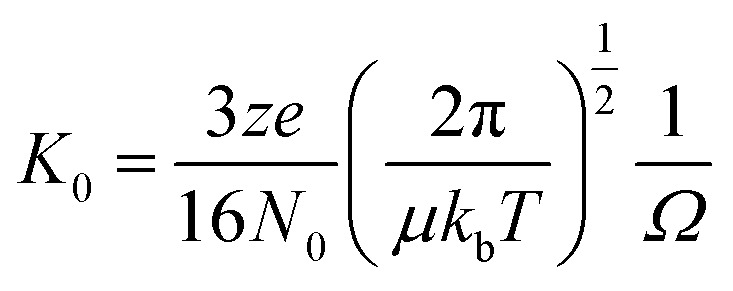
where *z* is the charge on the ion, *e* is the charge on an electron, *N*_0_ is the buffer gas number density, *μ* is the reduced mass of the buffer gas and ion, *k*_b_ is the Boltzmann constant, *T* is the effective temperature and *Ω* is the momentum transfer collision integral.

### Collision induced dissociation experiments

Collision induced dissociation experiments in an argon filled collision cell were performed using a QTOF Ultima (Micromass UK Ltd). The collision energy of the ions entering the collision cell was increased until the signal from the parent ion had been completely lost.

### FT-ICR MS experiments

High resolution mass spectrometry was performed on a modified 9.4 Tesla Apex Qe Fourier Transform Ion Cyclotron Resonance mass spectrometer (Bruker Daltonik GmbH). This provided ^13^C isotopic distributions which confirmed the identification of the peaks as isobaric aggromers or as conformers of the same species and also that the three disulphide bridges in insulin are all intact. The n-ESI source from the QTOF instrument was clamped in front of the cone and an external voltage applied to produce spray. The source accumulation time was 0.5 seconds, with the high frequency of 161282.396 Hz and low frequency of 24055.269 Hz. The voltages applied to the trap were 8.5 V on the entrance and 12 V on the exit. Data acquisition size was 1 048 576 bytes.

Calibration of 8 × 10^−10^ ppm (difference of 8 × 10^−4^ Da) accuracy was achieved using Bruker tune mix and data processed using DataAnalysis 4.0 (Bruker Daltonik GmbH). The simulated isotopic distributions were created from theoretical empirical formulas using the Simulate Isotopic Pattern function of DataAnalysis 4.0 (Bruker Daltonik GmbH).

### Molecular modelling

Molecular dynamics was performed using the AMBER10^13^ software package and monomer docking algorithms utilised the website Hex.^14^ Starting simulations employed an existing crystal structure of dimeric bovine insulin, PDB 2ZP6.^15^ Several multimeric species were constructed, from monomer up to hexamer, applying the crystallographic symmetry transformations given in the PDB file. After removing the zinc di-cations and submitting the structures to H++^16^ (imposing a pH of 2), the resulting ionic species were minimised *in vacuo*. CCS values were calculated using MOBCAL software^17^ implementing the trajectory method (TM). Results are given in ESI Table ST3.† Further details of all simulation methodology can be found in ESI.†

## Results and discussion

### Observations from mass spectrometry

**Fig. 1:**
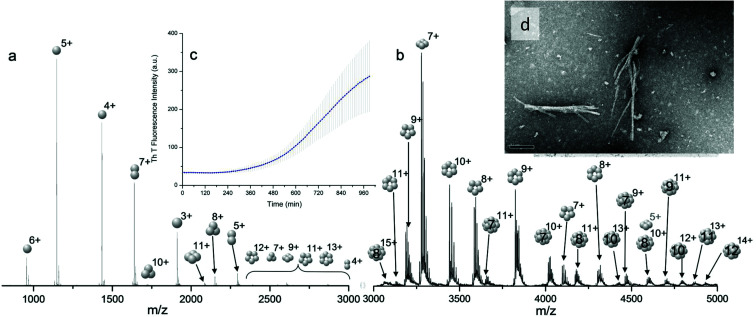
Mass spectrum of bovine insulin a, is a typical nano-electrospray (n-ESI) mass spectrum obtained from a solution of bovine insulin. b, Shows the higher *m*/*z* region. Oligomers of the form [*n*M + *z*H]^*z*+^ are observed with 1 ≤ *n* ≤ 15 and balls are used to represent the number of monomeric unit in each oligomer. c, Insert of the thioflavin T fluorescence data on insulin prepared in the same way and at the same concentration as for the mass spectrometry experiments. d, TEM image of fibrils of insulin formed under the same conditions, the scale bar is 1 μm.

Bovine insulin was studied by nano-electrospray mass spectrometry (n-ESI-MS) under conditions that favour protein aggregation, 523 μM at pH 2, but at room temperature, where aggregation takes place over a period of months rather than the hours required at elevated temperatures (Fig. 1). We have confirmed that fibrils are indeed formed under these solution conditions (by monitoring thioflavin T fluorescence (Fig. 1 insert c) and also imaging the resultant fibrils by transmission electron microscopy, Fig. 1 insert d) which are notably straight and rigid as well as often highly bundled. The mass spectrum (Fig. 1a and b) shows a wide distribution of multimeric and monomeric ions. The largest intensity species are attributed to three monomers of adjacent charge states; [M + 5H]^5+^, [M + 4H]^4+^ and [M + 3H]^3+^ at *m*/*z* 1147, 1434 and 1912 respectively. However there is also a significant contribution from peaks at *m*/*z* 1639, 2294 and 2868, where the principal contribution is from dimeric species (see below). Under electrospray conditions that best preserve oligomers, a predominant species is [2M + 7H]^7+^. When however, experimental conditions favour the break-up of aggregates, we observe an increase in the intensities of species assigned to [2M + 5H]^5+^ and [2M + 4H]^4+^ (see ESI Fig. S1 and S2†). It can be inferred from these data that [2M + 7H]^7+^ is significantly populated in solution and vulnerable to fragmentation, whereas the lower charge state dimers arise from fragmentation of larger species. Given that the dimer is a stable product ion and also a dominant species in the mass spectrum (Fig. 1a) we speculate that a dimer may be the core stacking unit in larger aggregates and therefore a key component of the fibrillogenesis process.

### Multiple oligomer conformations observed

**Fig. 2:**
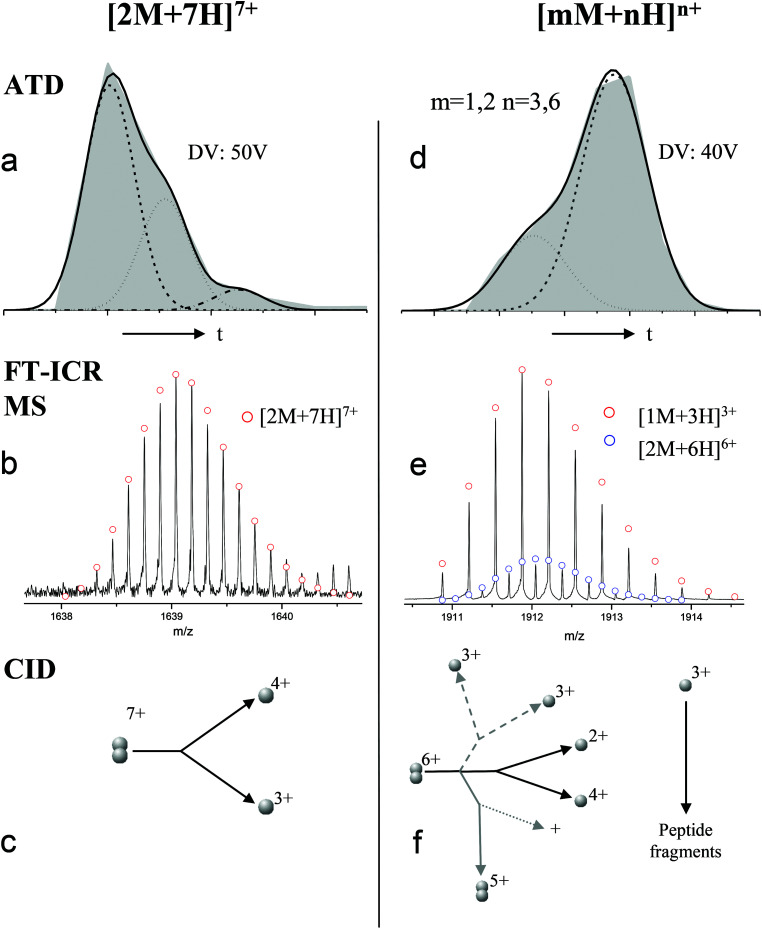
ATD (a and d), FTCR MS (b and e) and CID (c and f) comparison showing how FT-ICR MS and CID can distinguish between aggromers and conformations of the same species from multiple peaks present in the ATD. In the case of [2M + 7H]^7+^ multiple ATD peaks are assigned to distinct conformations since FT-ICR MS and CID data show no evidence for aggromer presence. Conversely for [M + 3H]^3+^, FT-ICR MS and CID data indicate the presence of the coincident [2M + 6H]^6+^ oligomer, thus explaining the observation of an earlier arriving species in the ATD. Dashed lines in CID data represent species which cannot be distinguished from the parent ion. Dotted lines represent unobserved species. Further details of the experimental conditions are found in ESI.† Again balls are used here simply to graphically represent the number of monomers in each oligomer.

For the oligomers of insulin, in several cases the ATDs show the presence of more than one species. As explained above, these could correspond either to multiple conformations of a single species or oligomers of a coincident *m*/*z* (which we term ‘aggromers’). We have used high resolution MS and collision induced dissociation (CID) to confirm assignment of these species. Fig. 2 shows two examples of species where multiple peaks were present in the ATD. In Fig. 2b the ion assigned as [2M + 7H]^7+^ gives an isotopic distribution consistent with the elemental composition of a dimer form of insulin (red circles show the predicted distribution). By contrast in Fig. 2e the species assigned nominally as [M + 3H]^3+^ shows an isotopic distribution of a monomer (red circles) and also a contribution from a species with twice the mass and charge (blue circles), which must be a dimer ([2M + 6H]^2+^). The ATD of this species (Fig. 2d) can therefore be conclusively assigned as containing dimer and monomer; the higher charge state of the dimer means it arrives sooner. Conversely the ATD of the [2M + 7H]^7+^ species (Fig. 2a) must be due to distinct conformational families, of which the most abundant arrives earlier and is thus the most compact. It was usually possible to resolve three families however on some occasions a fourth, weakly populated, family was observable. Our experimental timescale for IM-MS measurements is milliseconds and therefore these conformational families are stable for at least that time. The corresponding CCS values for [2M + 7H]^7+^ span an enormous conformational space from the principal conformer with a rotationally averaged CCS of 1217 Å^2^ to the least prevalent extended species with a CCS of 1701 Å^2^. The difference in the ratio of the intensities of the assigned *m*/*z* coincident species can be attributed to differences in the transfer optics of the two instruments (the IM-MS instrument possessing a source that has been optimised for the preservation of non-covalent interactions) and the differing timescales for analysis; milliseconds for IM-MS compared to seconds for FT-ICR MS.

These assignments are also confirmed by CID where mass selected ions are subjected to activation *via* collisions with argon, causing fragmentation. A comparison of what happens following CID is shown in Fig. 2c and 2f. For the [2M + 7H]^7+^ ion (Fig. 2c), only monomers are seen following CID, whereas for the nominally ‘monomeric’ [M + 3H]^+^ species (Fig. 2f), monomers are observed as fragment ions, which must come from a dimer parent, as previously identified *via* high resolution MS (Fig. 2e). Seven other oligomers; [2M + 9H]^9+^, [3M + 8H]^8+^, [3M + 10H]^10+^, [4M + 11H]^11+^, [5M + 11H]^11+^, [5M + 12H]^12+^ and [6M + 13H]^13+^ exhibit multiple peaks in their ATDs (ESI Fig. S7–12†). In all cases multiple peaks were found to be conformations of one species. A number of previous studies^18^ have indicated multiple peaks in ATDs of protein oligomers, however in this study the use of CID and FT-ICR is demonstrated that such assignments can be based on solid experimental evidence. This is unequivocal evidence for multiple, distinct, conformations of *oligomeric* species whose assignment has been achieved by further experiments. In every case the most compact conformation of the oligomer is present in the highest abundance, with two or three other larger conformers present. We have assigned CCS to every distinguishable conformer and multimer, from the four charge states seen for the monomeric form of insulin to the single charge state observable for the dodecamer (ESI Table ST1 and Fig. S13†). It is worth noting that the monomeric protein only presents single distinguishable conformers for every charge state.

Similar findings have been reported for monomeric peptides and proteins^19^ and for small amyloidogenic peptides^8^ but not for oligomers of amyloid forming proteins, where the charge state tends to be low compared with the mass. The presence of extended species in ATDs can be attributed to coulombic repulsion between the charges carried by the ions.^20^ De la Mora^21^ has derived an empirical relation for the maximum number of charges that can be present on the surface of a protein that still retains its native fold.2
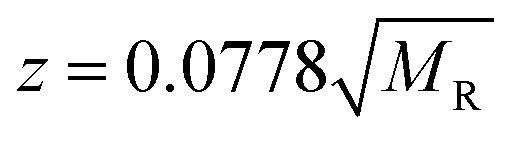
where *z* is the number of charges and *M*_R_ is the mass of the protein (or in this case, the oligomer). With the exceptions of the [M + 6H]^6+^ and [2M + 9H]^9+^ the observed charge on all insulin oligomer species is below the Rayleigh instability limit for its size (see ESI Fig. S14†), therefore the range of observed conformers for each oligomer (Fig. 2a and ESI Table ST1†) likely represents inherent structural variations present in solution rather than conformers arising from gas-phase coulombically driven unfolding. This explanation is supported by several studies that show insulin can adopt multiple conformations at low pH and high concentrations,^22^ and allows us to link the conformations we observe *via* IM-MS with atomistically resolved candidate geometries from MD simulations in implicit solvent. If we had found that the charges on insulin oligomers were above the instability limit, we would have been wary of extrapolating from gas-phase conformers to solution behaviour, and therefore recommend this approach.

**Fig. 3:**
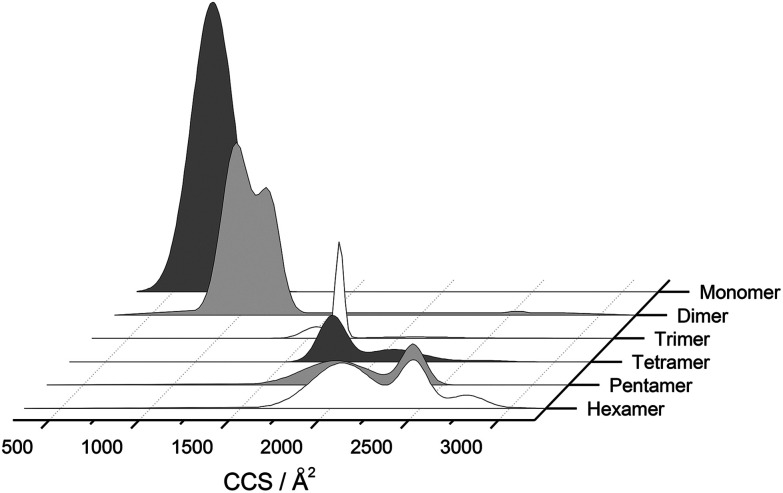
CCS Composite Plot. Plot of the conformational populations of each of the insulin oligomers as well as the monomer as measured using IM-MS. This data has been obtained from the areas of the deconvoluted peaks in the *m*/*z* selected ion ATDs. To aid interpretation, information from each charge state has been merged *via* a Gaussian fit to provide this composite plot, which highlights the separation capabilities of IM-MS. We have not included any higher order aggregates as they are of much lower intensity and could not easily be represented in this way.

The separation power of IM-MS is further demonstrated in Fig. 3. We are able to determine not only the size distribution of all species (*i.e.* monomer ions span collision cross sections of 704–978 Å^2^) but also unequivocally (coupled with high resolution FT-ICR MS, see above) assign sizes to oligomeric order (for example the ion at *m*/*z* 1639 can be uniquely identified as a dimer of CCS 1217 Å^2^). We have obtained CCS values for all of the oligomeric species observed (ESI Table S1†). These data can be directly compared to size exclusion chromatography (SEC). For example, Fink *et al.*^23^ used SEC to study insulin aggregation, assigning distinguishable ‘sizes’ to the hexamer, to compact and expanded dimers, and to compact, expanded, and unfolded monomer. With no mass information such data could be misinterpreted: Fig. 3 demonstrates that conformations occupied by the monomer and dimer populations significantly overlap, as do the conformations occupied by species including the trimer, tetramer, pentamer and hexamer. The CCS we assign somewhat under-represent the conformational spread of the discrete conformational families, (ESI Fig. S15 *cf.* Table ST1†), which we attribute to interconverting conformational families, stable on the timescale of our experiment, which for IM-MS are on the order of milliseconds.

### Effect of acidifying with HCl rather than formic acid

Fibrils grown in aqueous HCl (pH1.6) which has been used more commonly in the study of insulin aggregation, do exhibit different morphologies from those grown in Formic acid: such fibrils are on average longer and appear to form overlapping networks; short, thick bundles are not observed as frequently; several fibrils also appear noticeably curved (ESI Fig. S6a†). Therefore the properties of the acid do affect the morphology of the fibrillar products. The effect of different acids on the early steps of the assembly process is less conclusive. Ion mobility analysis of insulin acidified with HCl was greatly hindered by the formation of chloride adducts, clearly seen in mass spectra sprayed from these conditions (ESI Fig. S6b†), which had a deleterious effect on both the mass resolution and apparent sensitivity of the instruments. Therefore, determination of collision cross sections was impeded for most but the most intense aggregate species. The effect of using HCl instead of HCOOH as an acidifying agent had did not give rise to any discernible trend for monomeric ions (data not shown), with the 3+ monomer being more extended in HCl, but both 4+ and 5+ monomers appearing more compact when sprayed from HCl. Helium collision cross sections for insulin oligomers sprayed from hydrochloric acid also agree in general terms up to pentamers; some, potentially salient differences are noted. These include a shift in the cross sections for the dimeric ([2M + 5H]^5+^ and [2M + 7H]^7+^) ions towards higher values; in particular the [2M + 5H]^5+^ ion also displayed an additional feature arriving at longer times. Likewise, the [3M + 8H]^8+^ ion contained conformers that are significantly extended in HCl compared to the ones measured in HCOOH. The formation of these more extended configurations does not occur in larger oligomers detected; tetrameric ions ([4M + 9H]^9+^ and [4M + 10H]^10+^) are in fact found to be more compact in HCl.

### Molecular modelling of insulin monomers and dimers

MD simulations have been previously employed to examine the conformational flexibility of monomers of aggregating polypeptides.^7*b*,24,25^ Using our CCS data we have trained MD simulations to resolve the different conformations adopted by the dominant aggregate [2M + 7H]^7+^. Isotopic fits to the FT-ICR MS measurements (see for example Fig. 2b) confirm that the three disulfide bridges always remain in the oligomeric species, and this places important restrictions on the conformational space each oligomer can explore, a point which we will refer to later when we consider which oligomer conformation could compare best with the previously reported insulin protofilament.^26^

Following minimization of the crystal structure of dimeric bovine insulin, PDB 2ZP6^15^ the dimer has a calculated CCS of 1319 Å^2^, very similar to the measured CCS of one of the [2M + 7H]^7+^ conformers (1324 Å^2^). Thus, the second most populated gas-phase conformation occupies a volume comparable to that of insulin in its crystal structure.

The total charge of dimeric bovine insulin in solution at pH = 2 should be +8 if all of the acidic side chains are protonated. An insulin dimer extracted from the crystal structure PDB file yielded a net charge of +7 on submission to the webserver H++,^16^ consistent with the species observed by IM-MS, wherein one monomer carries a charge of +3 and the second a charge of +4 (as evidenced from the CID experiments). Glu4 from the A chain of one of the monomers is surrounded by many positively charged side chains and remained unprotonated. This facilitated further investigation of the very compact (1217 Å^2^) and highly extended (1709 Å^2^) conformations adopted by [2M + 7H]^7+^. The dimer was split into two monomeric species [M + 3H]^3+^ and [M + 4H]^4+^, and these were subjected to MD for up to 152 ns using the Amber10 software package,^13^ implementing the Amber ff99SB-ILDN force field^27^ and continuum solvation method.^28^ Further simulation details are found in ESI.†

**Fig. 4:**
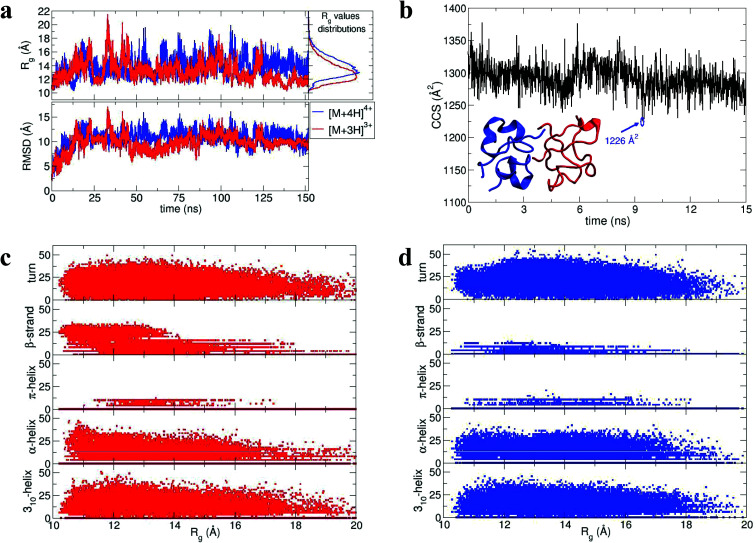
Dimer dynamics and correlation between size and secondary structure. The dimer was made up of a monomer [M + 4H]^4+^ and a monomer [M + 3H]^3+^ making a dimer [D + 7H]^7+^ to compare with experimental observations as described in the text and in ESI.† In all cases data from the [M + 4H]^4+^ is shown in blue and [M + 3H]^3+^ in red a, *R*_g_ and RMSD time series recorded along the 150 ns dynamics for the for [M + 4H]^4+^ and [M + 3H]^3+^. b, Gas phase dynamical evolution of the CCS for the most compact dimer. The structure with the lowest CCS is displayed. The experimental CCS for the most dominant, compact [2M + 7H]^7+^ species is 1217 ± 33 Å^2^. c and d, *R*_g_*versus* secondary structure percentage content for the constituent parts of the dimer, [M + 3H]^3+^ and [M + 4H]^4+^.

During dynamics, snapshots were collected every 2 ps to give an ensemble of 76 000 structures for each monomeric species. Both species sampled complex conformational landscapes: the radii of gyration (*R*_g_) span values between 10 and 22 Å, whereas the backbone root mean square deviations (RMSDs) from the crystal structure span values up to 15 Å (see Fig. 4a.) For the [M + 3H]^3+^ monomer, we have calculated both the *R*_g_ and the CCS for simulated insulin species, and find a linear correlation. This demonstrates that the two order parameters are comparable (for detailed information see Fig. S16 in ESI†). We have calculated the CCS of the lowest energy structures corresponding to the peak of the *R*_g_ distributions (Fig. 4a) obtaining: ∼962 Å^2^ and ∼998 Å^2^ for the [M + 3H]^3+^ and [M + 4H]^4+^ respectively (in agreement with experiment Table ST1†). As expected the [M + 4H]^4+^ species explores, on average, a slightly larger conformational space due to its higher charge.

To elucidate potential structures for the most abundant (and most compact) dimers observed, [M + 3H]^3+^ and [M + 4H]^4+^ species with the lowest calculated *R*_g_ were used in a protein–protein docking procedure implementing the webserver Hex,^14^ based on both shape and electrostatic correlation. After docking, the dimer with the lowest *R*_g_ was selected and subjected to dynamics *in vacuo* for 15 ns. The dynamical evolution of its CCS is reported in Fig. 4b. Despite the fact that our simulation time is short compared with the experimental timescale, and too short to effectively sample the conformational changes that occur to a desolvating protein,^29^ we find conformations consistent with the experimental result, as highlighted in Fig. 4b.

Across the dynamics trajectory a correlation between compactness and secondary structure exists which also holds true for the constituent monomers. Fig. 4c and 4d reports the scatter plot of *R*_g_ values *versus* helicity, beta and turn content, assessed through the DSSP algorithm.^30^ For both [M + 3H]^3+^ and [M + 4H]^4+^ the proportion of beta-strand secondary structure is notably higher for the smallest explored conformations, especially for the [M + 3H]^3+^ species. Quantitatively if *R*_g_ values are divided into quartiles, from the most compact to the most extended structures, the average percentages of β-strand content are 1.74, 0.88, 0.55 and 0.21 for [M + 4H]^4+^ and 14.09, 8.81, 5.63 and 3.04 for [M + 3H]^3+^. Other types of secondary structure are more equally distributed along the *R*_g_ values (Fig. 4c and d). It should be reiterated that the native state of insulin is predominantly α-helical, whereas the fibrillar form possesses β-strands and that – both experimentally and computationally – all of the disulphide bonds remain intact within the oligomers.

**Fig. 5:**
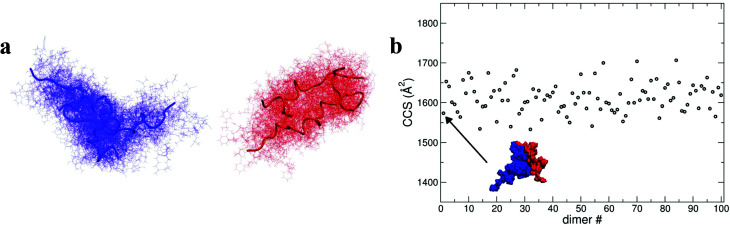
Structures and docking approaches for [2M + 7H]^7+^ a, representative structures (tubes) and conformations (lines) of the most populated clusters for [M + 4H]^4+^ and [M + 3H]^3+^. b, CCS values of the dimers derived from the protein–protein docking between the representative structures of the most populated families. The lowest energy “best docking” dimer is displayed with a surface representation.

As shown above, by selecting and docking the most compact monomers, MD simulations provide conformations of the insulin dimer that correspond to the compact species we observe experimentally. Similarly, by selecting and docking monomers with the highest *R*_g_ (see Fig. 4a) we can obtain dimers with CCS that match the experimental findings for the least compact forms. To model other stable forms of the insulin dimer a cluster analysis was performed^31^ on the monomeric species represented in Fig. 4a. Of the 10 conformational families (I–X) for both [M + 3H]^3+^ and [M + 4H]^4+^, the largest (I and II) each contain on the order of 25% of all the conformations sampled (see ESI section 4.2†). Representative structures of these most populated clusters (see Fig. 5a) were assembled using the same protein–protein docking approach. The resulting 1000 ‘best docking’ dimers were minimised *in vacuo* and the CCS calculated for the 100 lowest energy species. The corresponding values (Fig. 5b) distribute around a mean value of 1612 ± 38 Å^2^, close to the measured CCS for the third largest conformational family [CCS from experiment 1563 ± 10 Å^2^].

### Monomer contact interface and stability of docked dimers

To analyse the contact interface between dimers assembled using the docking approach, the distribution of the α-carbon pairwise (CA–CA) distances over the 1000 docked structures were calculated. We assign the notation *m*_*i*_^(*j*)^ to the monomeric units, where *i* = I, II, …, X characterises a representative structure of one among the ten clusters reported earlier. Only the first two most populated clusters have been considered in this case *i.e. i* = I and II. The notation *j* represents the considered net charge, *i.e. j* = 3 or 4. The representative structure of the most populated cluster was assembled with itself and with a representative from the second most populated, obtaining the following 6 families each comprising 1000 dimers: *m*_I_^(3)^*m*_I_^(3)^, *m*_I_^(3)^*m*_II_^(3)^, *m*_I_^(4)^*m*_I_^(3)^, *m*_I_^(4)^*m*_II_^(3)^, *m*_I_^(4)^*m*_I_^(4)^, *m*_I_^(4)^*m*_II_^(4)^.

The resulting distributions are projected onto an *xy* plane, where *x* and *y* are the numbered sequences of the first and second monomer respectively (Fig. S17†). This procedure enables us to select the lowest energy dimers from the most frequently represented monomer–monomer interface. The stability of each dimer was evaluated *via* a Molecular Mechanics Poisson Boltzmann Surface Area (MM-PBSA) calculation implemented in the Amber software package.^32^ Dimers were immersed in a box of TIP3P^33^ water molecules and a neutral charge achieved by the addition of chloride ions. Simulation details are given in ESI.†

**Fig. 6:**
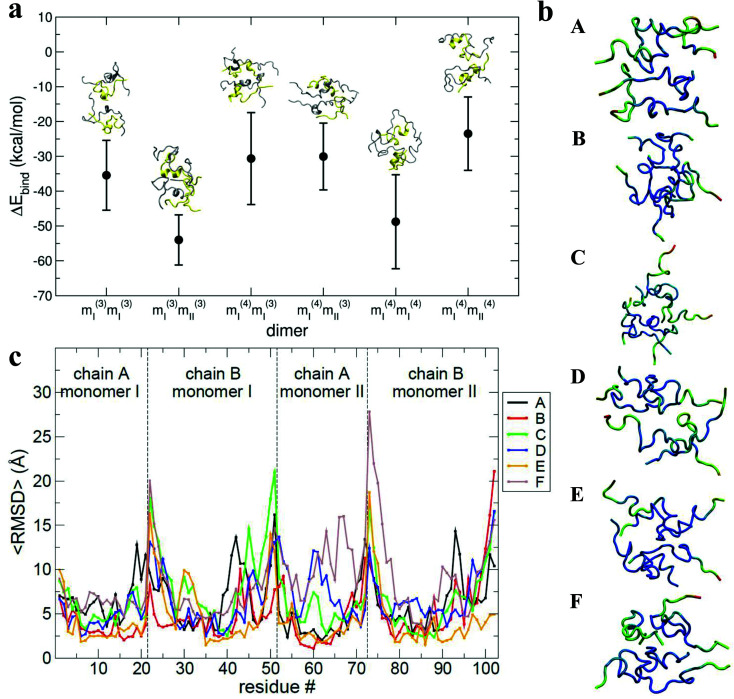
a, Values of binding energy between monomers forming the selected dimers. The binding energy calculated *via* MM-PBSA for each selected dimer (*m*_*i*_^(*j*)^*m*_*i*_^(*j*)^) is depicted along with the final structure for each from the MD trajectory. A and B chains are yellow and silver respectively. b, Structures with residues coloured to show the 〈RMSD〉 where blue is lowest, green intermediate and red largest. Letters A, B, C, D, E and F in the legends box refer to the selected dimers from the families *m*_I_^(3)^*m*_I_^(3)^, *m*_I_^(3)^*m*_II_^(3)^, *m*_I_^(4)^*m*_I_^(3)^, *m*_I_^(4)^*m*_II_^(3)^, *m*_I_^(4)^*m*_I_^(4)^, and *m*_I_^(4)^*m*_II_^(4)^ respectively. c, Residue number *versus* the 〈RMSD〉. Lines marked with letters A, B, C, D, E and F in the legend box refer to selected dimers from families *m*_I_^(3)^*m*_I_^(3)^, *m*_I_^(3)^*m*_II_^(3)^, *m*_I_^(4)^*m*_I_^(3)^, *m*_I_^(4)^*m*_II_^(3)^, *m*_I_^(4)^*m*_I_^(4)^, and *m*_I_^(4)^*m*_II_^(4)^ respectively.

The results of the calculations are shown in Fig. 6a: all of the binding free energies are negative, testifying that the dimers derived from protein–protein docking are stable. Furthermore the more compact dimers are the most stable ones with the most negative binding energies. This correlates with our experimental observation of the most compact dimers being the most abundant. Analysis of the contributions to the binding energy indicates that in all six cases hydrophobic interactions add favourably to the driving force for self-assembly (see Table ST4 in ESI†). This does not hold true for the hydrophilic interactions, which are negative in only two out of six cases. This confirms that hydrophobic interactions are fundamental in stabilising the assembly of the monomeric units. Even though the docking procedure was performed using monomers unfolded in implicit solvent, and hence likely to favour surface-exposed hydrophilic residues and burial of hydrophobic groups, the MM-PBSA procedure has revealed the importance of the hydrophobic interactions in the monomer contact interface.

The interaction interface is retained during each 25 ns simulation, as shown by the residue specific average root mean square deviation (〈RMSD〉) value (Fig. 6b and c). The highest values of 〈RMSD〉 are from residues that do not participate in the interface, these are located at the termini of both the chains, although there is greater variability in the B chain. The interface between monomers in the different dimers is not identical, with both A–B/A–B and A–B/B–A stacking mechanisms observed.

During the dimer dynamics 25 structures were saved (one every nanosecond), from which the water molecules were stripped out. These structures were then minimised *in vacuo* and their CCS calculated. Values between ∼1500 and ∼1700 Å^2^ were obtained, (Fig. S18†) again correlating well with experimental values.

### Can we relate prefibrillar insulin oligomers to fibrillar architecture?

Using low resolution 3-D structures of insulin fibrils, Jiménez *et al.*^26^ postulate that the single protofilament of insulin consists of stacked monomers, and further that each insulin molecule occupies two layers, where the A and B chains of insulin stack on top of each other, connected by the native disulphide bridges. From the given electron densities of the single protofilament they estimate a dimension of (30 × 40) Å^2^.^26^ If we assume that the inter layer spacing is less than the smallest dimension, which it must be if the layers are connected by disulphide bridges, we can obtain a value for *R*_g_ of ∼19.5 Å for the smallest unit that forms these protofilaments. From the relationship we have derived between *R*_g_ and CCS (Fig. S16†), we can compare this to our measured values. The most extended +3 monomer from our implicit solvent calculations has an *R*_g_ of 21.4 Å and a corresponding CCS of ∼1391 Å^2^. The average values of *R*_g_ for monomers are 13.2 Å (CCS 1120 Å^2^), and 13.9 Å (CCS 1160 Å^2^), for the +3 and +4 species respectively (Fig. 4a). In our ion mobility measurements, no monomeric species has a CCS that exceeds 1000 Å^2^, and the most populated are less than this (Fig. 3 and Table ST1†) in contrast to these values from our calculation. The dimeric species we observe experimentally have CCS values that are much more comparable to the *R*_g_ inferred from the data of Jiménez *et al.*^26^ and interestingly, the most populated dimeric conformers (Fig. 3) cover a range of *R*_g_ from ∼17.5–22. It is tempting to speculate that structures in the gas phase, where the dielectric constant is 1, may be more similar to those present in the hydrophobic environment of the protofilaments.^34^ Indeed our data show that a dimeric building block cannot be ruled out, and that if as postulated by Jiménez *et al.*^26^ the protofilament is comprised of stacked monomers, these must elongate substantially as the fibrils grow. IM-MS shows that monomers only exist as a single conformational family, whereas higher order oligomers have several stable conformers over the millisecond IM-MS experimental timescale, which may reveal the extended forms required for fibril growth. In comparing our findings with the model of Bleiholder *et al.*^8^ for isotropic *versus* fibril growth in peptide oligomers, it must be noted that insulin is significantly larger than any of the small model peptides in that work and that we observe it in many more charge states, and therefore the relationship between CCS and the occurrence of beta sheets in any given aggregating oligomer system may well not be the same. This said our finding are in broad agreement with that work, indicating that both isotropic and potential fibril forming growth pathways can be observed with ion mobility mass spectrometry.

**Fig. 7:**
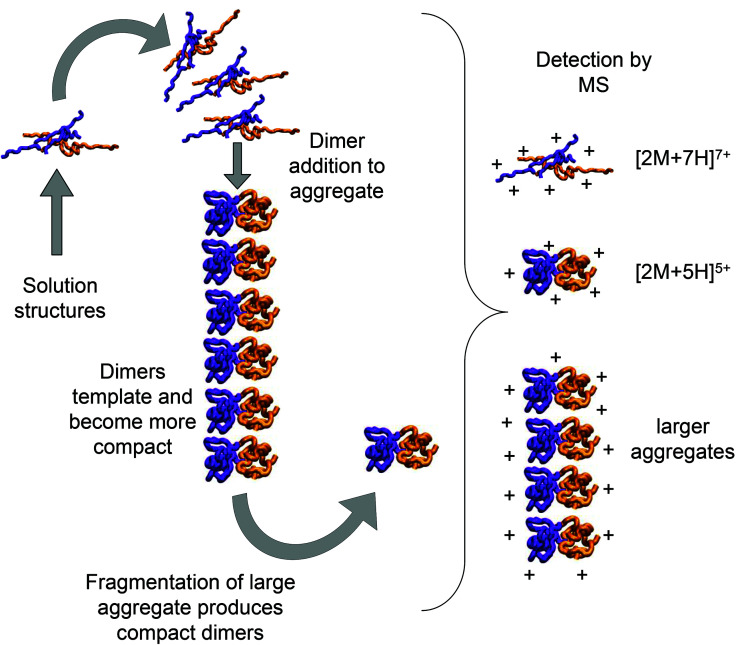
Schematic of a possible insulin dimer assembly mechanism informed by experiment. Mass spectrometry and molecular modelling have shown that extended dimeric structures which form [2M + 7H]^7+^ ions in are present in solution. We speculate here that these could form the core stacking unit in small prefibrilar oligomers, which would be detectable by mass spectrometry and may also fragment to form more compact templated dimers detected as [2M + 5H]^5+^ ions. [2M + 5H]^5+^ and [2M + 7H]^7+^ structures are generated by molecular modelling as described above and rendered with VMD software.

As we show above, under electrospray conditions which best preserve oligomers, a predominant species is [2M + 7H]^7+^. When however, experimental conditions favour the break-up of aggregates, we observe an increase in the intensities of species assigned to [2M + 5H]^5+^ and [2M + 4H]^4+^ and it can be inferred that [2M + 7H]^7+^ is significantly populated in solution, whereas the lower charge state dimers arise from fragmentation of larger species. Given that the dimer is a stable product ion and also a dominant species in the mass spectrum we speculate that a dimer may be the core stacking unit in larger aggregates and therefore a key component of the fibrillogenesis process. A schematic of such a possible assembly mechanism is given in Fig. 7. The question then arises as to why other higher order oligomers are observed? An explanation is to attribute these lag phase species as off-pathway species or kinetic traps rather than necessarily on-pathway species. We then can speculate that: (a) the elongated dimer is the only, or a dominant member of the subset of growth competent species and (b) that growth *may* proceed by the addition of the dimeric species rather than by monomer addition and (c) this *may be* because the dimer is found in an elongated conformation.

## Conclusion

Using ion mobility mass spectrometry we observe multiple conformations of oligomers present before the onset of accelerated fibril growth. The identities of these conformations have been definitively confirmed by FT-ICR MS and are stable for milliseconds (IM-MS timescale) to seconds (FT-ICR MS timescale). This indicates that these species have a sufficiently high energy barrier to prevent interconversion during measurement. Modelling, trained by IM-MS measurements, has been used to characterise aggregates in atomistic detail. The most dominant conformational species for each aggregate is always the most compact form, which modelling suggests, in the case of the dimer, to be enriched in β sheet secondary structure. Dimers of this form would fit to the electron density previously found in the single protofilaments of insulin.

## Supplementary Material

AN-140-C5AN01253H-s001

## References

[cit1] Serio T. R., Cashikar A. G., Kowal A. S., Sawicki G. J., Moslehi J. J., Serpell L., Arnsdorf M. F., Lindquist S. L. (2000). Nucleated conformational conversion and the replication of conformational information by a prion determinant. Science.

[cit2] Knowles T. P. J., Waudby C. A., Devlin G. L., Cohen S. I. A., Aguzzi A., Vendruscolo M., Terentjev E. M., Welland M. E., Dobson C. M. (2009). An Analytical Solution to the Kinetics of Breakable Filament Assembly. Science.

[cit3] Nettleton E. J., Tito P., Sunde M., Bouchard M., Dobson C. M., Robinson C. V. (2000). Characterization of the oligomeric states of insulin in self-assembly and amyloid fibril formation by mass spectrometry. Biophys. J..

[cit4] Nielsen L., Khurana R., Coats A., Frokjaer S., Brange J., Vyas S., Uversky V. N., Fink A. L. (2001). Effect of environmental factors on the kinetics of insulin fibril formation: Elucidation of the molecular mechanism. Biochemistry.

[cit5] Adams M. J., Blundell T. L., Dodson E. J., Dodson G. G., Vijayan M., Baker E. N., Harding M. M., Hodgkin D. C., Rimmer B., Sheat S. (1969). Structure of Rhombohedral 2 Zinc Insulin Crystals. Nature.

[cit6] Fabris D., Fenselau C. (1999). Characterization of allosteric insulin hexamers by electrospray ionization mass spectrometry. Anal. Chem..

[cit7] Bernstein S. L., Dupuis N. F., Lazo N. D., Wyttenbach T., Condron M. M., Bitan G., Teplow D. B., Shea J. E., Ruotolo B. T., Robinson C. V., Bowers M. T. (2009). Amyloid-beta protein oligomerization and the importance of tetramers and dodecamers in the aetiology of Alzheimer's disease. Nat. Chem..

[cit8] Bleiholder C., Dupuis N. F., Wyttenbach T., Bowers M. T. (2011). Ion mobility-mass spectrometry reveals a conformational conversion from random assembly to beta-sheet in amyloid fibril formation. Nat. Chem..

[cit9] Young L. M., Cao P., Raleigh D. P., Ashcroft A. E., Radford S. E. (2014). Ion mobility spectrometry-mass spectrometry defines the oligomeric intermediates in amylin amyloid formation and the mode of action of inhibitors. J. Am. Chem. Soc..

[cit10] Beveridge R., Phillips A. S., Denbigh L., Saleem H. M., MacPhee C. E., Barran P. E. (2015). Relating gas phase to solution conformations: Lessons from disordered proteins. Proteomics.

[cit11] McCullough B. J., Kalapothakis J., Eastwood H., Kemper P., MacMillan D., Taylor K., Dorin J., Barran P. E. (2008). Development of an ion mobility quadrupole time of flight mass spectrometer. Anal. Chem..

[cit12] Harvey S. R., MacPhee C. E., Barran P. E. (2011). Ion mobility mass spectrometry for peptide analysis. Methods.

[cit13] Case D. A. , Darden T. A., Cheatham III T. E., Simmerling C. L., Wang J., Duke R. E., Luo R., Crowley M., Walker R. C., Zhang W., Merz K. M., Wang B., Hayik S., Roitberg A., Seabra G., Kolossvry I., Wong K. F., Paesani F., Vanicek J., Wu X., Brozell S. R., Steinbrecher T., Gohlke H., Yang L., Tan C., Mongan J., Hornak V., Cui G., Mathews D. H., Seetin M. G., Sagui C., Babin V. and Kollman P. A., Amber 10, University of California, San Francisco, 2008

[cit14] Macindoe G., Mavridis L., Venkatraman V., Devignes M. D., Ritchie D. W. (2010). HexServer: an FFT-based protein docking server powered by graphics processors. Nucleic Acids Res..

[cit15] Jaimohan S. M. , Naresh M. D. and Mandal A. B., Crystal structure of Bovine Insulin, 2008

[cit16] Anandakrishnan R., Aguilar B., Onufriev A. V. (2012). H++ 3.0: automating pK prediction and the preparation of biomolecular structures for atomistic molecular modeling and simulations. Nucleic Acids Res..

[cit17] Mesleh M. F., Hunter J. M., Shvartsburg A. A., Schatz G. C., Jarrold M. F. (1996). Structural information from ion mobility measurements: Effects of the long-range potential. J. Phys. Chem..

[cit18] Kloniecki M., Jablonowska A., Poznanski J., Langridge J., Hughes C., Campuzano I., Giles K., Dadlez M. (2011). Ion Mobility Separation Coupled with MS Detects Two Structural States of Alzheimer's Disease A beta 1–40 Peptide Oligomers. J. Mol. Biol..

[cit19] Pierson N. A., Chen L., Valentine S. J., Russell D. H., Clemmer D. E. (2011). Number of Solution States of Bradykinin from Ion Mobility and Mass Spectrometry Measurements. J. Am. Chem. Soc..

[cit20] Clemmer D. E., Jarrold M. F. (1997). Ion mobility measurements and their applications to clusters and biomolecules. J. Mass Spectrom..

[cit21] Fernandez de la Mora J. (2000). Electrospray ionization of large multiply charged species proceeds via Dole's
charged residue mechanism. Anal. Chim. Acta.

[cit22] Ahmad A., Uversky V. N., Hong D., Fink A. L. (2005). Early events in the fibrillation of monomeric insulin. J. Biol. Chem..

[cit23] Ahmad A., Millett I. S., Doniach S., Uversky V. N., Fink A. L. (2003). Partially folded intermediates in insulin fibrillation. Biochemistry.

[cit24] Porrini M., Zachariae U., Barran P. E., MacPhee C. E. (2013). Effect of Protonation State on the Stability of Amyloid Oligomers Assembled from TTR(105-115). J. Phys. Chem. Lett..

[cit25] Baumketner A., Bernstein S. L., Wyttenbach T., Bitan G., Teplow D. B., Bowers M. T., Shea J. E. (2006). Amyloid beta-protein monomer structure: A computational and experimental study. Protein Sci..

[cit26] Jiménez J. L., Nettleton E. J., Bouchard M., Robinson C. V., Dobson C. M., Saibil H. R. (2002). The protofilament structure of insulin amyloid fibrils. Proc. Natl. Acad. Sci. U. S. A..

[cit27] Lindorff-Larsen K., Piana S., Palmo K., Maragakis P., Klepeis J. L., Dror R. O., Shaw D. E. (2010). Improved side-chain torsion potentials for the Amber ff99SB protein force field. Proteins.

[cit28] Feig M., Onufriev A., Lee M. S., Im W., Case D. A., Brooks C. L. (2004). Performance comparison of generalized born and Poisson methods in the calculation of electrostatic solvation energies for protein structures. J. Comput. Chem..

[cit29] Breuker K., McLafferty F. W. (2008). Stepwise evolution of protein native structure with electrospray into the gas phase, 10(-12) to 10(2) S. Proc. Natl. Acad. Sci. U. S. A..

[cit30] Kabsch W., Sander C. (1983). Dictonary of Protein Secondary Structure - Pattern Recognition of Hydrogen-Bonded and Geometrical Features. Biopolymers.

[cit31] Shao J. Y., Tanner S. W., Thompson N., Cheatham T. E. (2007). Clustering molecular dynamics trajectories: 1. Characterizing the performance of different clustering algorithms. J. Chem. Theory Comput..

[cit32] Kollman P. A., Massova I., Reyes C., Kuhn B., Huo S., Chong L., Lee M., Lee T., Duan Y., Wang W., Donini O., Cieplak P., Srinivasan J., Case D. A., Cheatham T. E. (2000). Calculating Structures and Free Energies of Complex Molecules: Combining Molecular Mechanics and Continuum Models. Acc. Chem. Res..

[cit33] Jorgensen W. L., Chandrasekhar J., Madura J. D., Impey R. W., Klein M. L. (1983). Comparison of Simple Potential Functions for Simulating Liquid Water. J. Chem. Phys..

[cit34] Barran P. E., Polfer N. C., Campopiano D. J., Clarke D. J., Langridge-Smith P. R. R., Langley R. J., Govan J. R. W., Maxwell A., Dorin J. R., Millar R. P., Bowers M. T. (2005). Is it biologically relevant to measure the structures of small peptides in the gas-phase?. Int. J. Mass Spectrom..

