# Supplementary material for: Early stages of insulin fibrillogenesis examined with ion mobility mass spectrometry and molecular modelling
Source: Analyst. 2015 Sep 9;140(20):7000–11. doi: 10.1039/c5an01253h (PMC11226345; doi:10.1039/c5an01253h)
Supplement: AN-140-C5AN01253H-s001 [file AN-140-C5AN01253H-s001.pdf]

Electronic Supplementary Material (ESI) for Analyst.
This journal is © The Royal Society of Chemistry 2015

Supplementary Information for

Early stages of insulin fibrillogenesis examined with ion mobility mass spectrometry and molecular modelling

Authors:

Harriet Cole[#†], Massimiliano Porrini[†], Ryan Morris[†], Tom Smith[#], Jason Kalapothakis[#†], Stefan Weidt[#], C. Logan Mackay[#], Cait E. MacPhee[†] & Perdita E. Barran*[1]

Author Affiliations

[#]EastChem School of Chemistry, Joseph Black Building, The King's Buildings, West Mains Rd, Edinburgh EH9 3JJ

[†] SUPA, School of Physics and Astronomy, James Clark Maxwell Building, The King's Buildings, West Mains Rd, Edinburgh EH9 3JZ

[*]Michael Barber Centre for Collaborative Mass Spectrometry, School of Chemistry, Manchester Institute of Mass Spectrometry, The University of Manchester, Manchester, M1 7DN

Supplementary Information 1
1. Supplementary MS and IM-MS data 3
1.1 Cone Voltage Experiments 3
1.2 Concentration Experiments 5
1.3 Injection Energy Studies 6
ATD, FTICR MS and CID data 8
2. Supplementary Structural Characterisation 15
2.1 Collision Cross Sectional Values 15
2. 2 Graph of CCSs 17
2.4 Number of Charges on Protein Surface 18
3. Experimental Methodology 18
3.1 Fitting of ATD peaks 19
3.2 Source Conditions 21
3.3 Thioflavin T binding assay 21
3.4 Transmission Electron Microscopy 22
4. Simulation Methodology 22
4.1 Molecular Modelling 22
4.2 Monomeric species $[M+3H]^{3+}$ and $[M+4H]^{4+}$ 22
4.3 Correlation between CCS and R_g 23
4.4 Contact interface and stability of dimers derived from docking 24
4.4.1 Breakdown of the contributions to the binding energy of representative dimers 25
4.4.2 CCSs of dimer structures from dynamics in water solvent 26
5. References 26

1. Supplementary MS and IM-MS data

1.1 Cone Voltage Experiments

Mass spectra of insulin were taken at different cone voltages. When the cone voltage is increased; aggregate species are observed to break up.

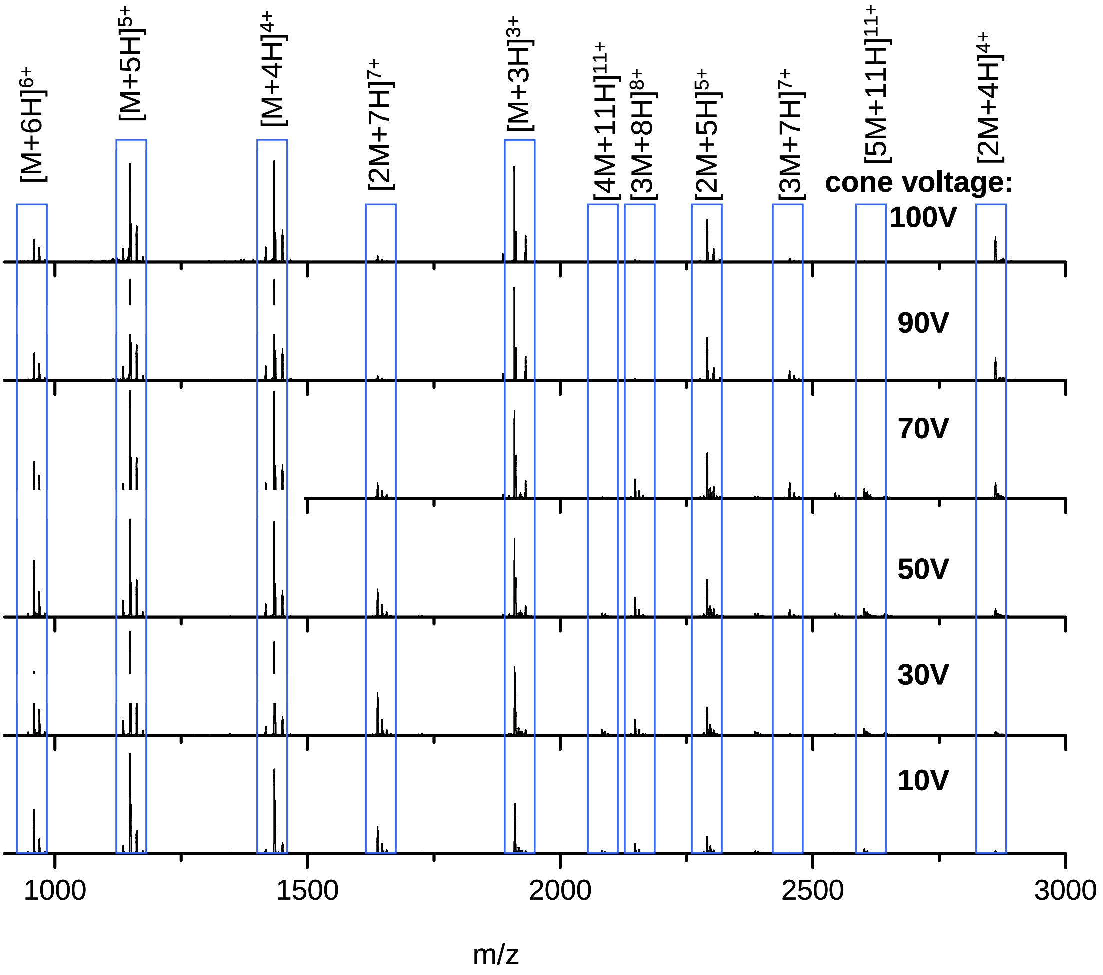

Figure S1 | Spectra at increasing cone voltages between 10V and 100V showing oligomer population changes.

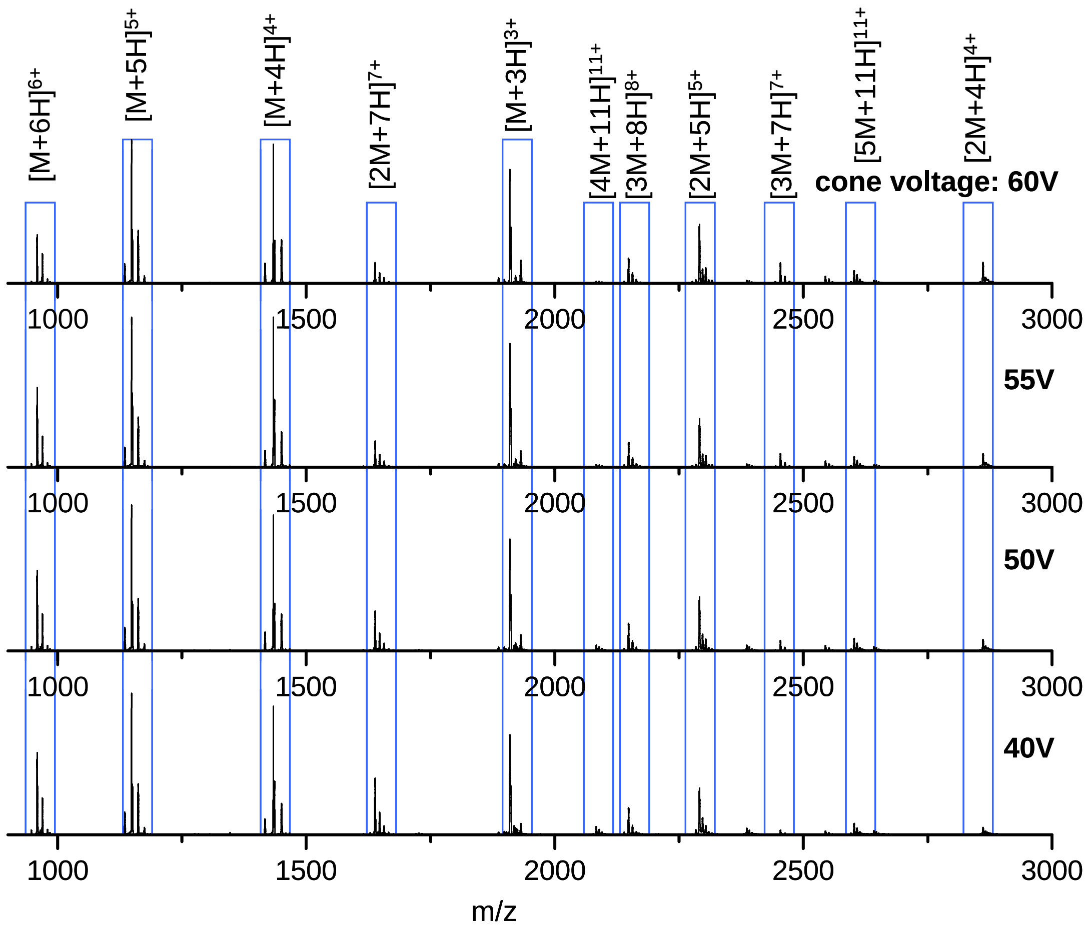

Figure S2 | Spectra at increasing cone voltages enlarged to show in detail the range between 40V and 60V. The peak corresponding to $[2M+7H]^{7+}$ decreases with the increase in cone voltage, conversely $[2M+5H]^{5+}$ increases in abundance as cone voltage is increased.

No appreciable dissociation of the multimers takes place at voltages below 60V. All IM-MS experiments were conducted with a cone voltage of 50V, thus below the limit at which voltages dissociate the oligomers. Further experiments were performed lowering the pressure in the source of the mass spectrometer. These conditions favourably fragment the higher order oligomers.
Similar trends; a decrease in intensity of $[2M+7H]^{7+}$ and an increase in the relative population of $[2M+5H]^{5+}$ are observed.

1.2 Concentration Experiments

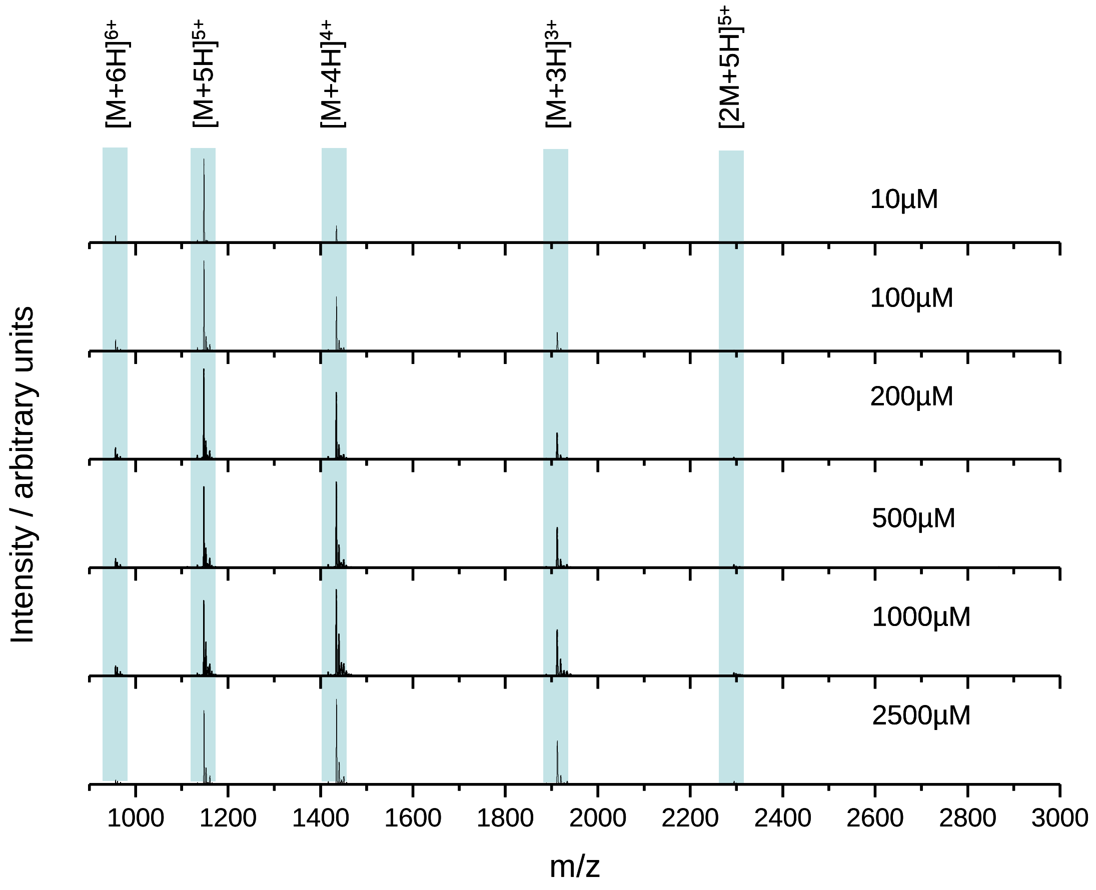

Figure S3 | Spectra at increasing concentrations enlarged to show in detail the *m/z* region between 900 and 3000. The dominant species in the majority of spectra is $[M+4H]^{4+}$. An exception to this is for the lower concentrations of insulin where $[M+5H]^{5+}$ is of the highest intensity. At higher concentrations, there is an increase the number and population of larger oligomers observed. An enlargement of the higher m/z region for formic acid solution conditions allows greater discernment.

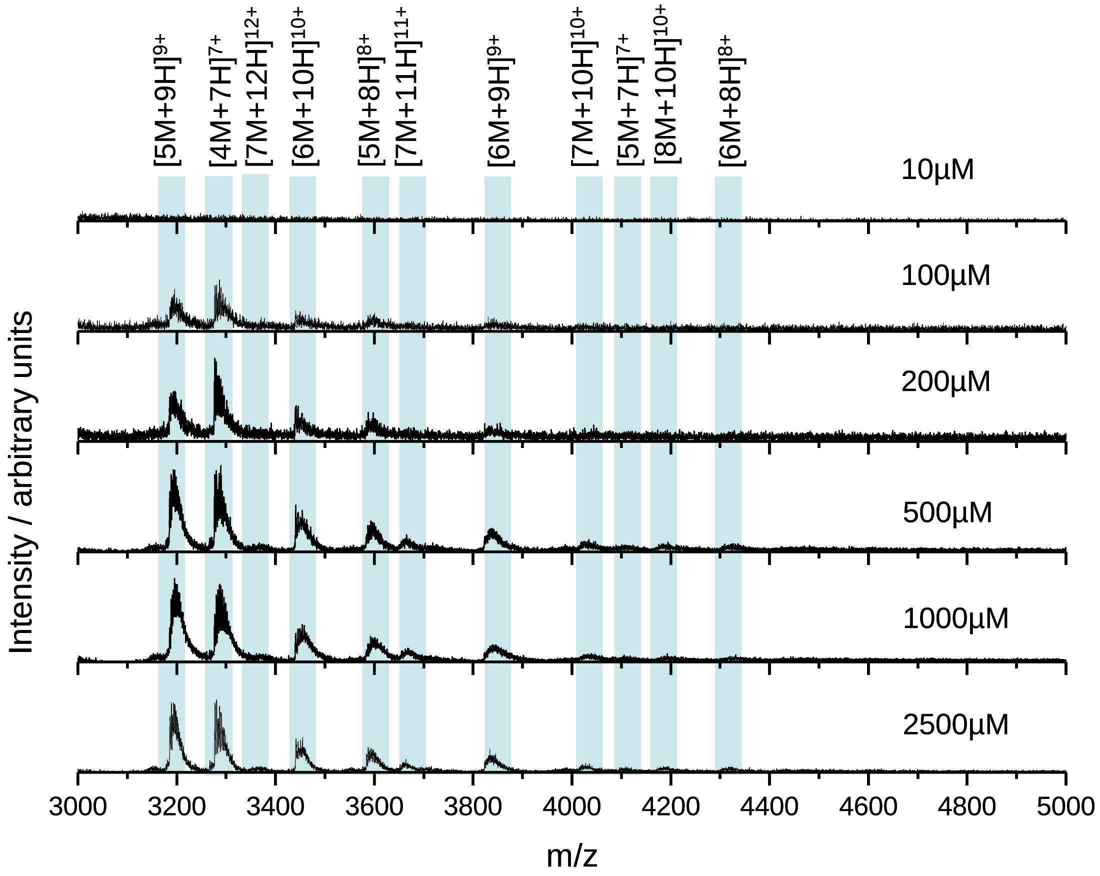

Figure S4 | Spectra at increasing insulin concentration enlarged to show in detail the *m/z* region between 3000 and 5000. At 500µM and above a wide range of larger oligomers are present at significant intensities. Higher order oligomer populations remain quantitatively similar with increasing concentrations after a threshold of 500µM has been reached. A solution of aqueous formic acid containing insulin at a concentration of 523µM was chosen for the majority of further experiments.

1.3 Injection Energy Studies

Injection energy studies were carried out to ensure that the extended oligomer conformers observed were not caused by structural unfolding in the gas phase due to activation of the ions by high-energy collisions as they are transported in the mass spectrometer. IM-MS data was acquired whilst varying the voltage that injects the ions into the drift tube, known as the injection energy. An set of ATDs of the $[2M+7H]^{7+}$ are shown below, as this is one of the oligomeric species which possesses extended conformers.

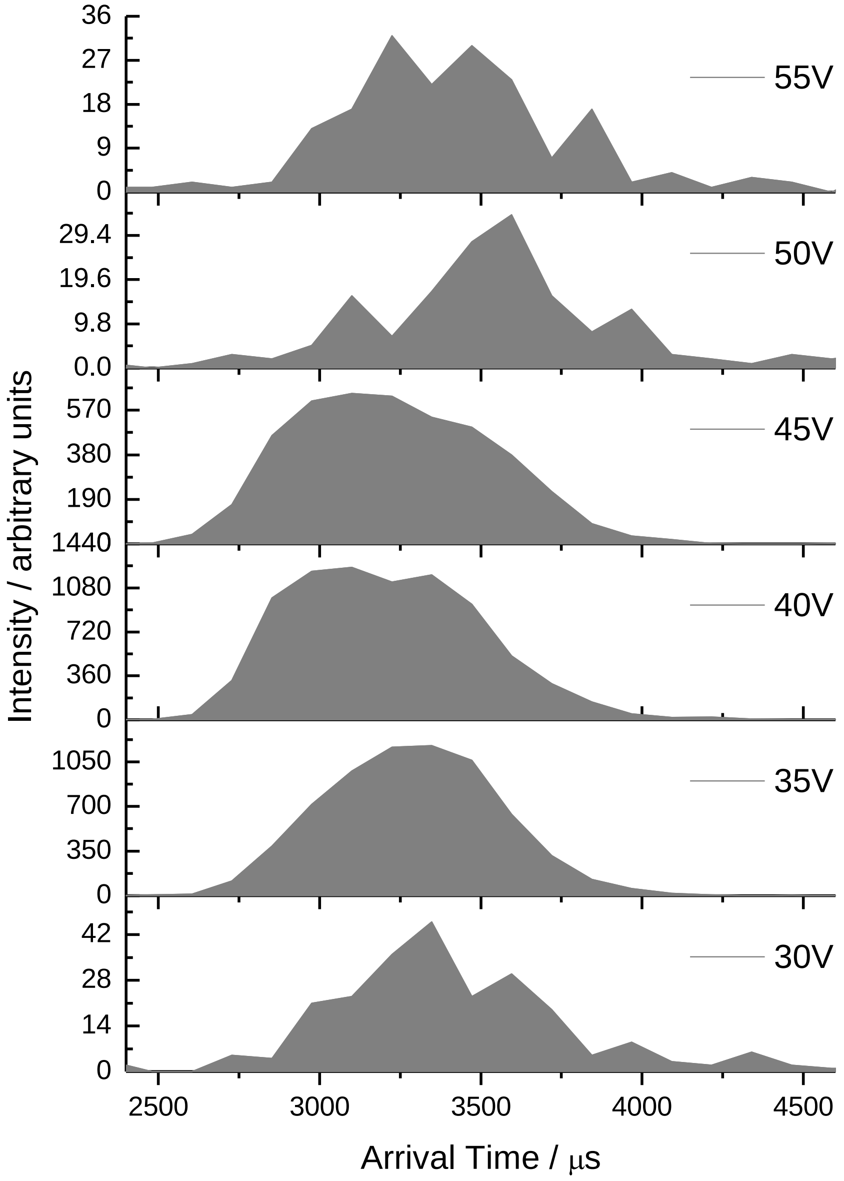

Figure S5 | [2M+7H]$^{7+}$ ATDs acquired at a range of injection energies.

1.4 Acidifying with HCl

a

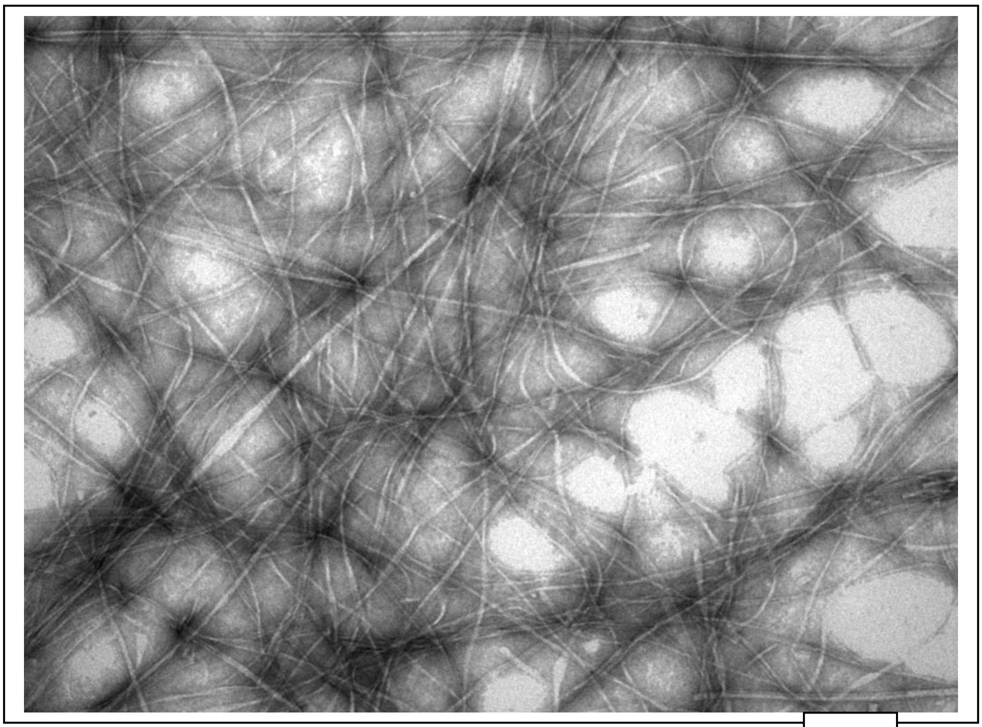

b

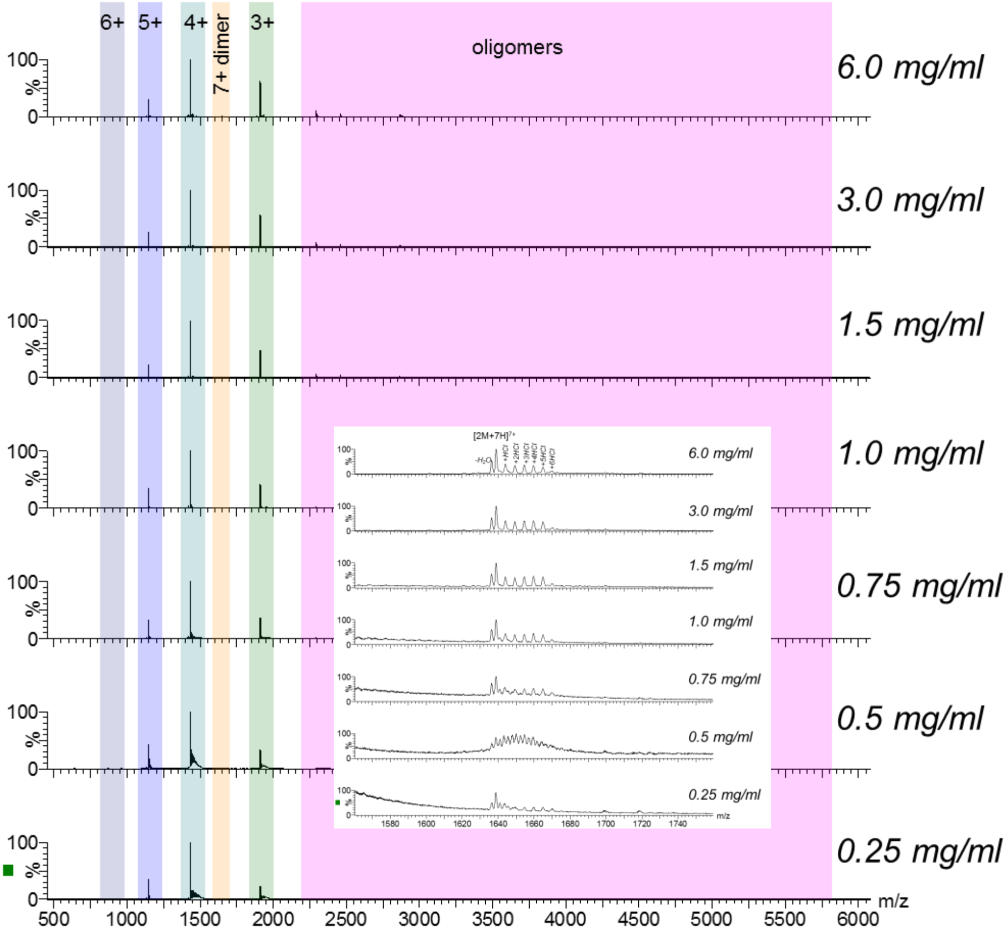

Figure S6 | A TEM image of 4mg/ml insulin solution, incubated over 3 days at 60°C in solutions acidified with HCl

B nESI Mass Spectra at increasing insulin concentration when it has been acidified with HCl . At 500µM and above a wide range of larger oligomers are present at significant intensities. The insert shows the extent of aductation due to chloride species.

1.5 ATD, FTICR MS and CID data

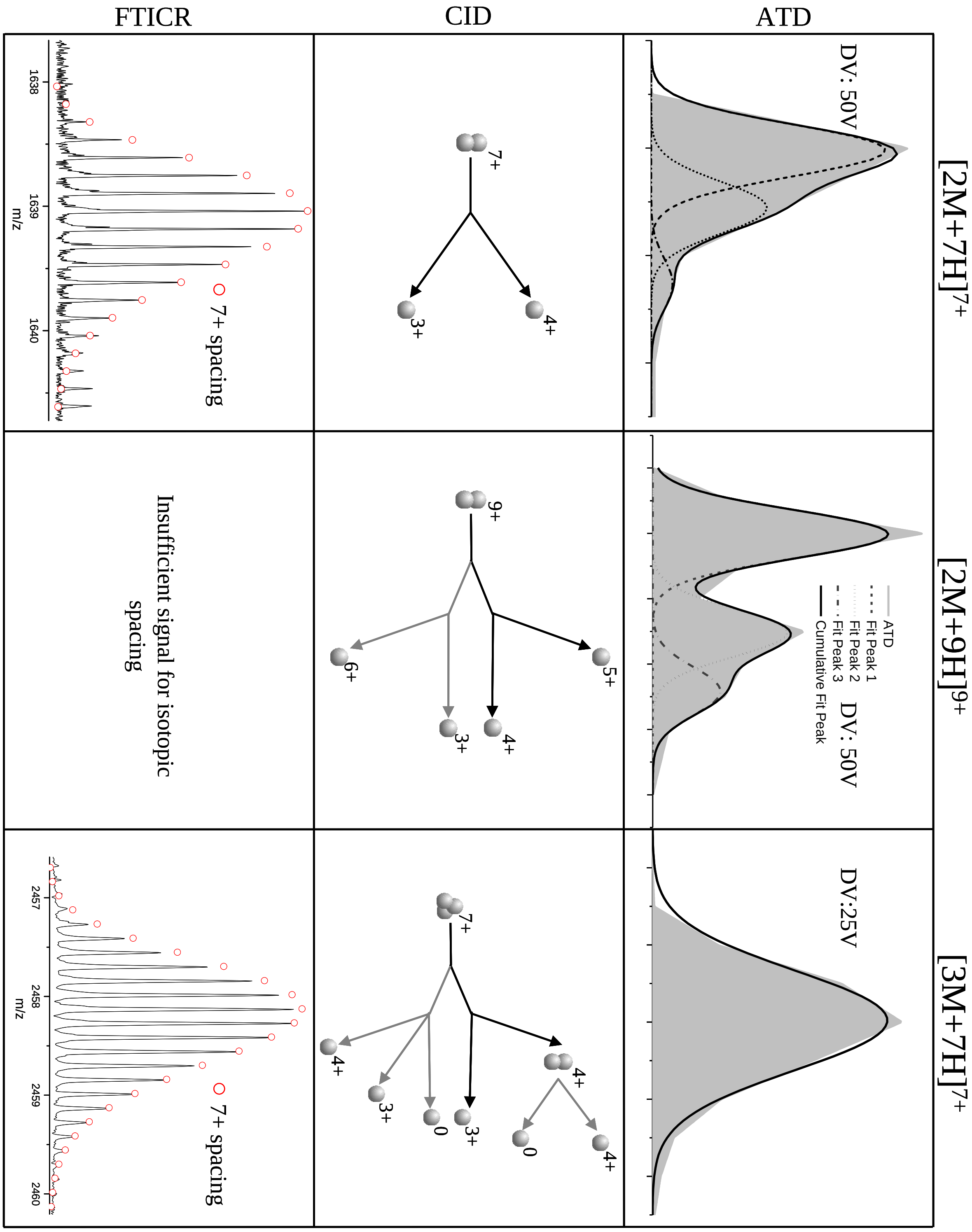

Figure S7 | ATD, FT-ICR MS and CID data for $[2M+7H]^{7+}$, $[2M+9H]^{9+}$ and $[3M+7H]^{7+}$. For CID data black lines denote the dominant fragmentation pathway.

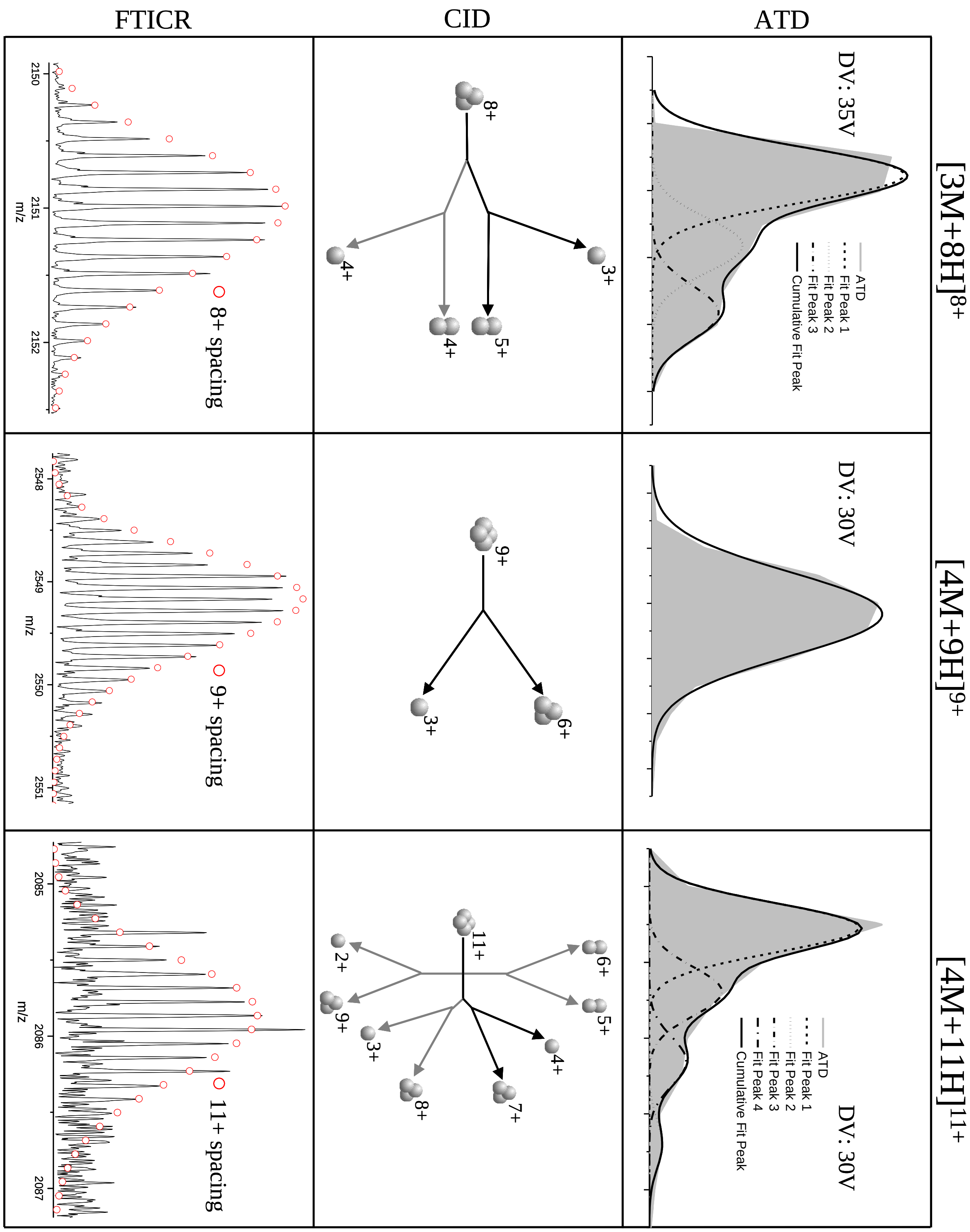

Figure S8 | ATD, FT-ICR MS and CID data for $[3M+8H]^{8+}$, $[4M+9H]^{9+}$ and

[4M+11H]$^{11+}$. For CID data black lines denote the dominant fragmentation pathway.

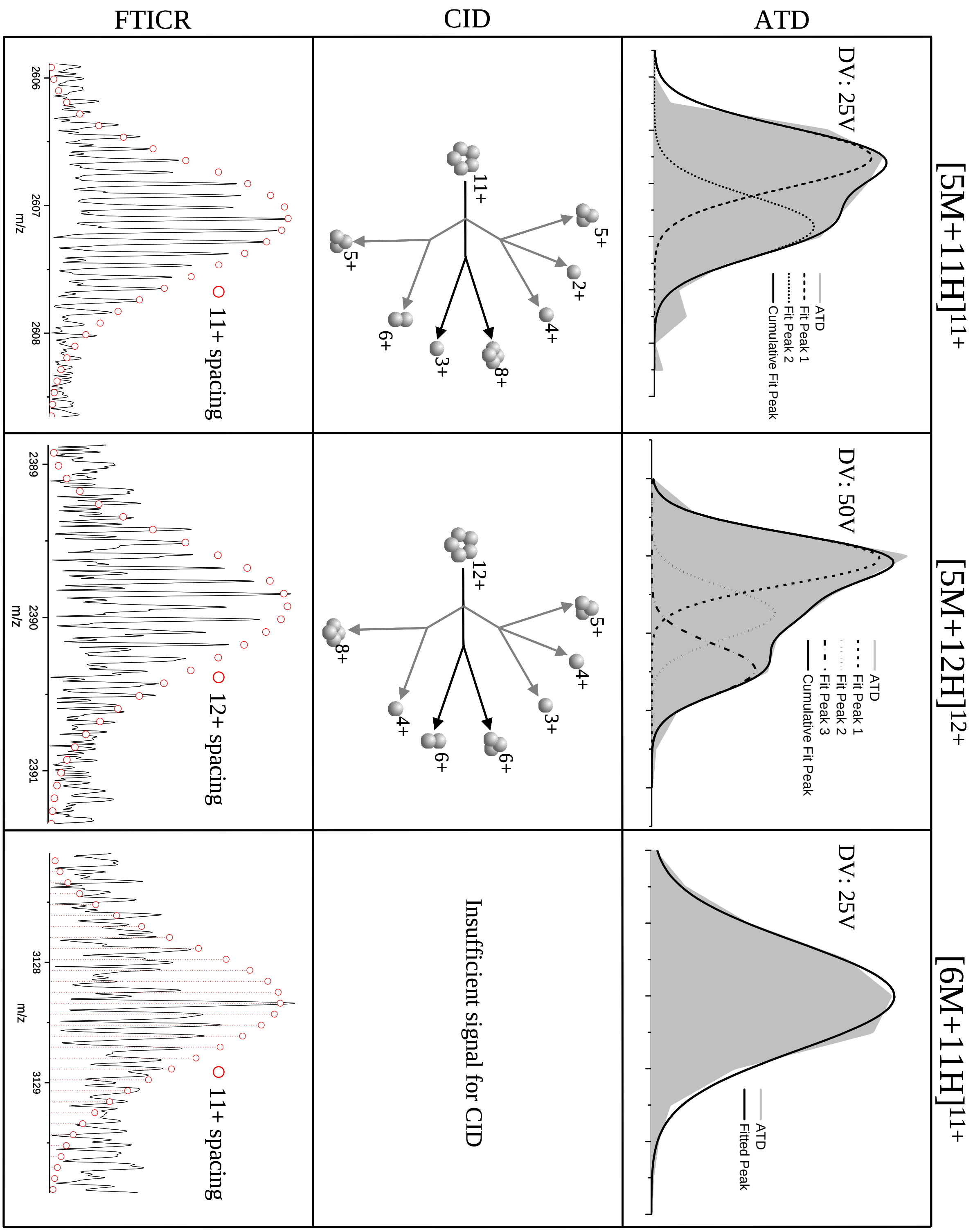

Figure S9 | ATD, FTICR MS and CID data for [5M+11H]$^{11+}$, [5M+12H]$^{12+}$ and [6M+11H]$^{11+}$. For CID data black lines denote the dominant fragmentation pathway.

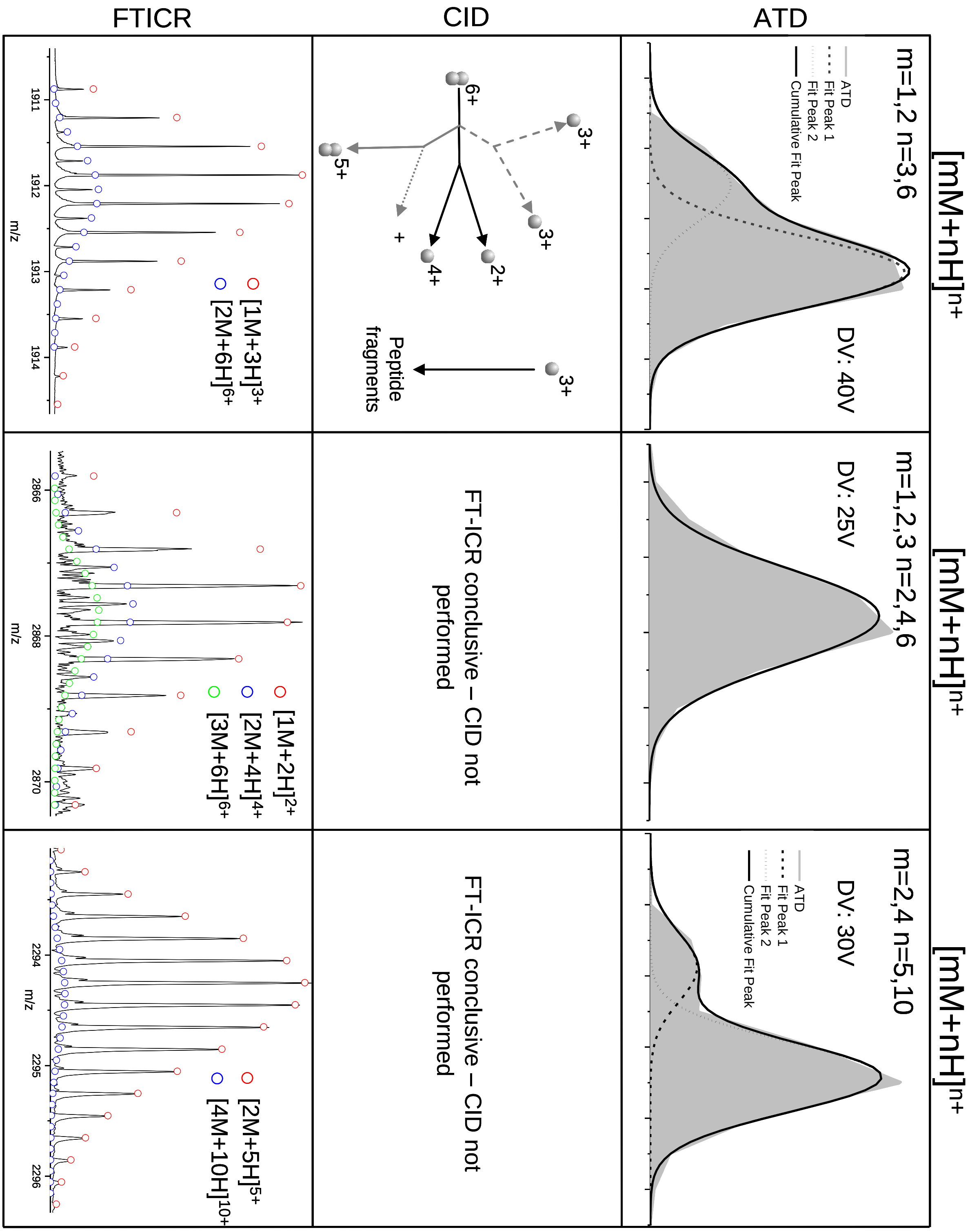

Figure S10 | ATD, FTICR MS and CID data for $[mM+nH]^{n+}$, where m=1,2,3 and n=3,6,9, m=1,2,3 and n=2,4,6 and m=2,4 and n=5,10. For CID data black lines denote the dominant fragmentation pathway. Dotted lines denote fragments which cannot be detected by MS. Dashed lines indicate fragments which have the same m/z as parent ions.

The $[2M+7H]^{7+}$ ATDs are shifted to higher arrival times only when the injection voltage is higher than 45V. All IM-MS experiments were performed with an injection energy of 39.7V

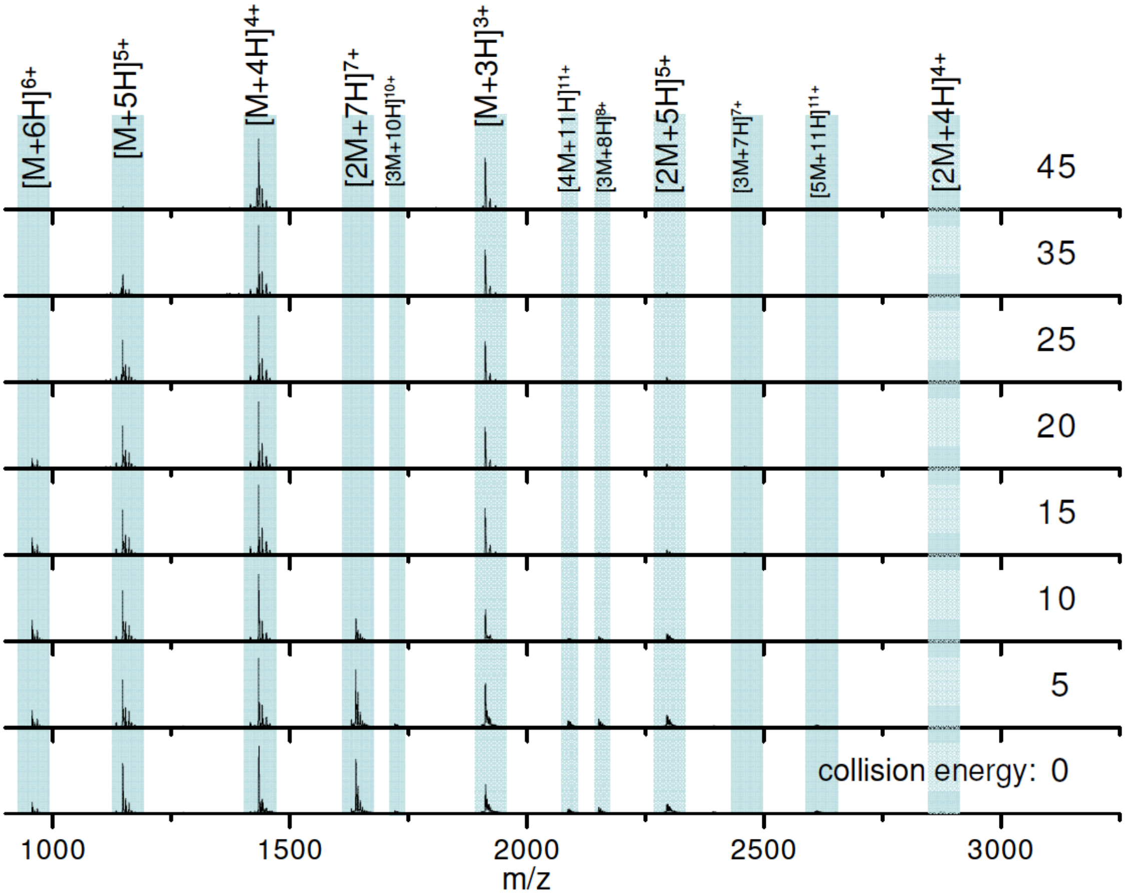

Figure S11 Mass spectra taken at increasing collision energy showing the change in the population of oligomers.

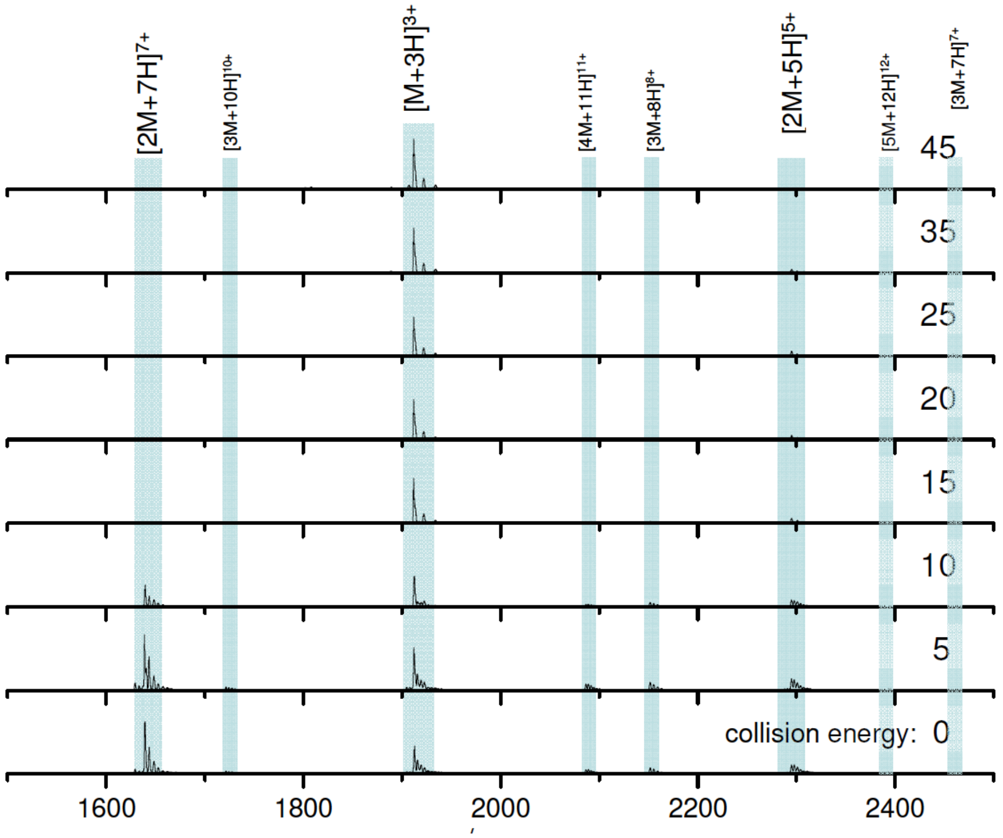

Figure S12 Zoom of data shown in Figure S11 to show in detail the m/z region between 1500 and 2500.

2. Supplementary Structural Characterisation

2.1 Collision Cross Sectional Values

	CCS / Å²	
**Species**	**Average**	**Standard Deviation**
**$[M+6H]^{6+}$**	978.9	22.2
**$[M+5H]^{5+}$**	912.3	5.6
**$[M+4H]^{4+}$**	805.7	4.3
**$[M+3H]^{3+}$**	704.5	24.1
**$[2M+9H]^{9+}$**	1678.9	179.1
**$[2M+9H]^{9+}$**	2305.3	250.5
**$[2M+9H]^{9+}$**	2565.2	71.6
**$[2M+7H]^{7+}$**	1217.0	32.5
**$[2M+7H]^{7+}$**	1323.7	49.3
**$[2M+7H]^{7+}$**	1563.9	9.6
**$[2M+7H]^{7+}$**	1701.0	*
**$[2M+6H]^{6+}$**	1095.6	20.1

**$[2M+5H]^{5+}$**	1200.9	9.0
**$[2M+4H]^{4+}$**	961.3	17.0
**$[3M+10H]^{10+}$**	1609.2	333.0
**$[3M+10H]^{10+}$**	1722.9	35.4
**$[3M+10H]^{10+}$**	2560.9	136.6
**$[3M+8H]^{8+}$**	1621.9	97.7
**$[3M+8H]^{8+}$**	1764.3	48.1
**$[3M+8H]^{8+}$**	1948.3	*
**$[3M+7H]^{7+}$**	1682.1	14.6
**$[4M+11H]^{11+}$**	1954.2	47.9
**$[4M+11H]^{11+}$**	2363.6	107.3
**$[4M+11H]^{11+}$**	2731.0	7.2
**$[4M+11H]^{11+}$**	3034.8	*
**$[4M+10H]^{10+}$**	1907.1	84.5
**$[4M+ 9H]^{9+}$**	2160.4	102.6
**$[4M+7H]^{7+}$**	1754.2	40.7
**$[5M+12H]^{12+}$**	2249.5	49.6
**$[5M+12H]^{12+}$**	2622.0	4.7
**$[5M+12H]^{12+}$**	2911.3	107.5
**$[5M+12H]^{12+}$**	3418.7	*
**$[5M+11H]^{11+}$**	2259.4	64.3
**$[5M+11H]^{11+}$**	2516.9	30.5
**$[5M+11H]^{11+}$**	2877.9	*
**$[5M+9H]^{9+}$**	2265.7	35.0
**$[5M+8H]^{8+}$**	1982.8	57.4
**$[5M+7H]^{7+}$**	1680.3	8.3
**$[6M+13H]^{13+}$**	2207.9	148.5
**$[6M+13H]^{13+}$**	2663.4	108.7
**$[6M+13H]^{13+}$**	2997.8	80.2
**$[6M+13H]^{13+}$**	3246.5	*
**$[6M+13H]^{13+}$**	3673.1	*
**$[6M+11H]^{11+}$**	2695.9	86.1
**$[6M+10H]^{10+}$**	2465.2	41.2
**$[6M+9H]^{9+}$**	2205.2	26.2
**$[6M+8H]^{8+}$**	1888.6	62.3
**$[7M+13H]^{13+}$**	3113.1	64.7
**$[7M+12H]^{12+}$**	2938.0	66.6
**$[7M+11H]^{11+}$**	2791.2	68.2
**$[7M+10H]^{10+}$**	2399.6	82.6
**$[8M+15H]^{15+}$**	3608.7	102.6
**$[8M+13H]^{13+}$**	3198.7	60.7
**$[8M+11H]^{11+}$**	2575.3	63.6
**$[9M+14H]^{14+}$**	3476.1	29.5
**$[9M+13H]^{13+}$**	3146.3	62.0
**$[11M+17H]^{17+}$**	4134.9	46.7
**$[12M+17H]^{17+}$**	4040.2	36.3

Table ST1 | Rotationally averaged collision cross sections for all multimeric species and conformations observed. The collision cross sections presented are the average of three repeats with the associated standard deviation. * represents species which were only identified in one repeat and therefore do not have an associated standard deviation.

2. 2 Graph of CCSs

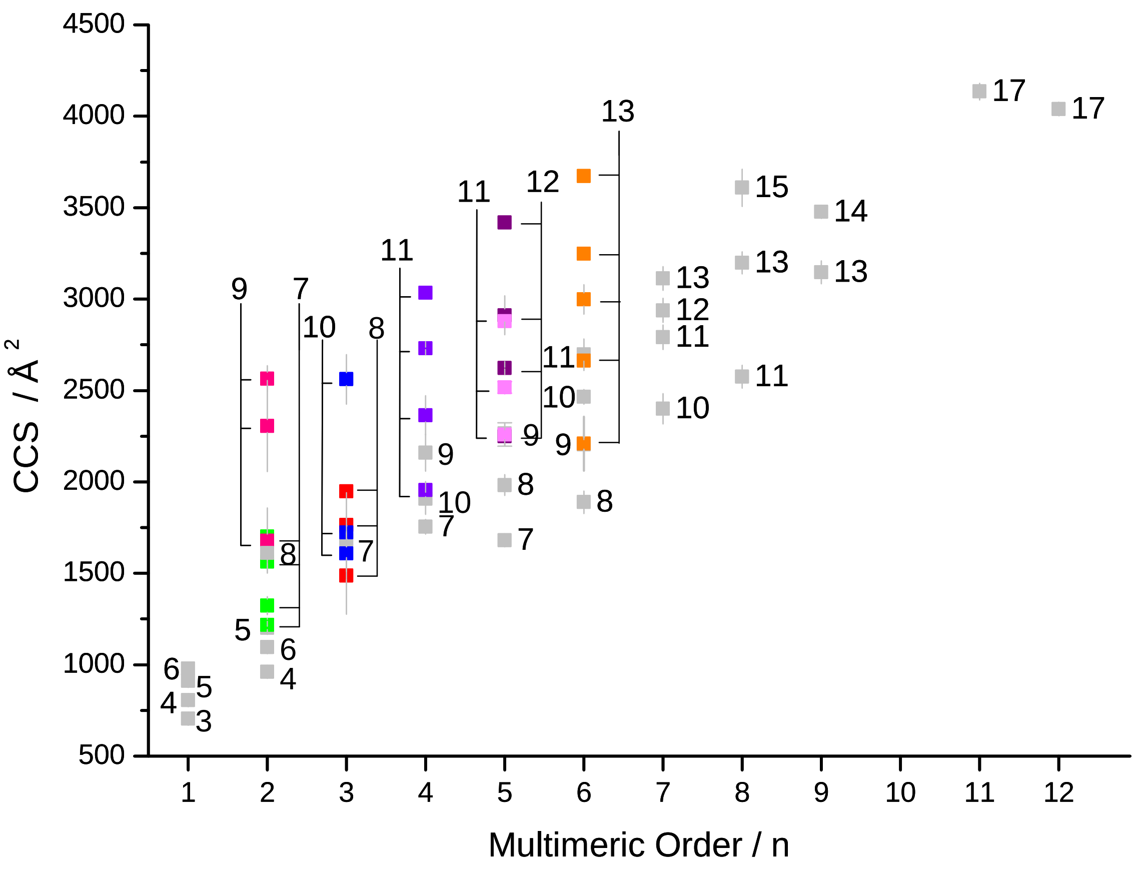

Figure S13 | Graph of multimeric order versus CCS for 523µM insulin in aqueous pH2 formic acid. Labels are the associated charge state of each oligomer. Error bars represent standard deviations of three repeated measurements.

2.4 Number of Charges on Protein Surface

A large number of charges will increase the CCS of an aggregate due to coulombic repulsion [1,2]. From de la Mora's empirical relation for the maximum number of charges on the surface of a protein which has retained its native fold (see main text), the graph below plots this against the experimentally observed charge states for different oligomeric orders. All of the observed species carry more charges than the multimeric order of the oligomer.

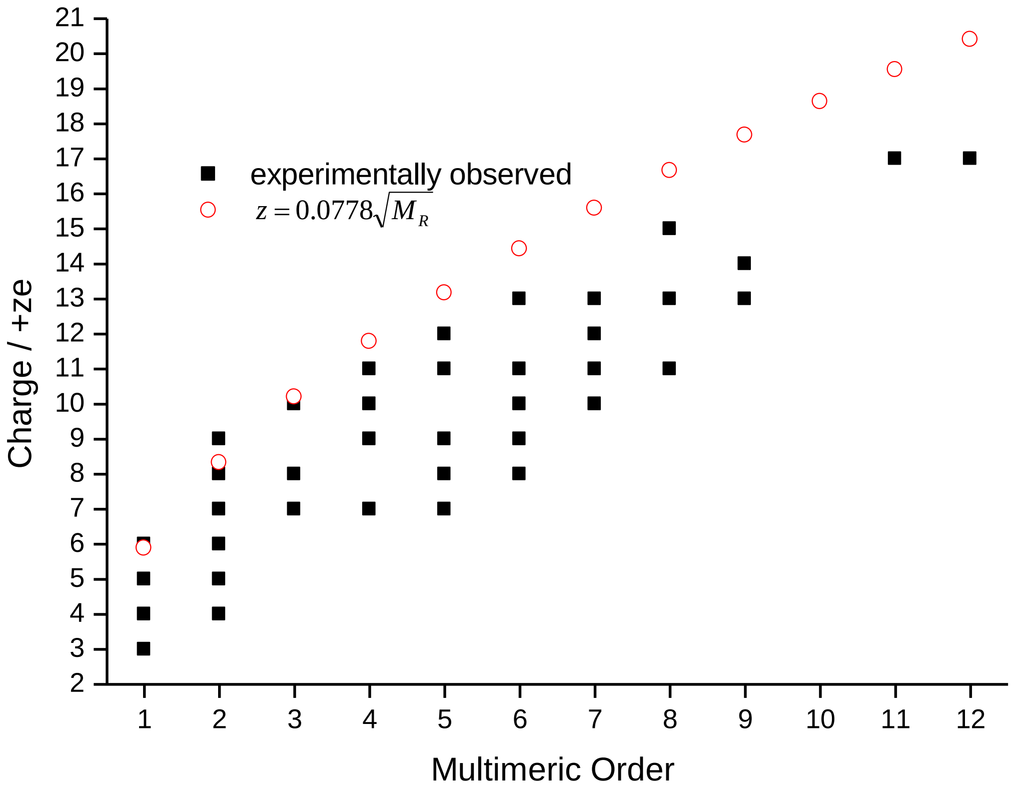

Figure S14 | de la Mora relation The observed charge carried by each oligomer compared to the de la Mora relation for the maximum number of charges able to be carried on the surface of a protein which has retained its native fold.

The observed charge on all oligomeric species is consistent with the surface protonation of a compact globular species, with exceptions $[M+6H]^{6+}$ and $[2M+9H]^{9+}$. Therefore differences in the charge oligomers carry can be attributed to inherent structural variations present in solution and not charge driven coulombic unfolding. A comparison between the experimentally determined CCSs of insulin oligomers and those of other globular proteins of comparable mass further supports this as they are very similar. A similar argument has been put forward by Smith *et al.* [3].

3. Experimental Methodology

3.1 Fitting of ATD peaks

From the experimental ATDs the presence of multiple species coincident in *m/z* space can be conferred. Additional information can be obtained by comparing the experimentally determined ATDs with those predicted from models of ion transport. Moseley *et al.* [4] have shown that the axial ion current $J(x, t)$ generated from an infinitely thin disk of ions drifting with constant velocity and diffusing in a drift tube is given by:

$$J(x,t)=\frac{ae^{-\frac{(x-vt)^2}{4D_L t}}}{4\sqrt{\pi D_L t}}\left(v+\frac{x}{t}\right)\left(1-e^{-\frac{r_0^2}{4D_T t}}\right) \qquad \text{Equation} \qquad \text{S2}$$

where t is the drift time, x is the drift length, r_0 the radius of the entrance aperture, v the drift velocity, D_L and D_T the longitudal and transverse diffusion coefficients, respectively and a a scaling factor which depends on the initial amount of ions and also their transmission efficiency through the instrument. Two parameters in this expression are of particular significance: the drift velocity and the longitudal diffusion coefficient. The drift velocity is given by

$$v=KE \qquad \text{Equation.} \qquad \text{S3}$$

K being the ionic mobility. At low field strength the diffusion coefficient and the mobility are related via the Nernst-Einstein-Townsend relation:

$$K=\frac{zeD}{k_B T} \qquad \text{Equation} \qquad \text{S4}$$

Thus, at least in theory, longitudal diffusion coefficients and drift speeds can be fitted to a single parameter. Nevertheless, in the data collected for insulin and insulin multimer ions sprayed from acidified solutions this is seldom the case. The peaks in the arrival time spectrum are consistently broader than predicted from the Nernst-Einstein-Townsend relation. This fact is illustrated in Figure S9. A number of factors not taken into account with this model can contribute to the observed widening of the ATD. The input pulse width is one example; nevertheless that alone cannot explain the observed widths since the initial pulse cannot be spread over an interval greater than 40µs; however ATD widths observed are of the order of milliseconds; therefore a difference greater than 5% cannot be due to the initial pulse width. Another factor that is very difficult to account for is the distortion of the longtitudinal ion distribution in regions of the instrument outside the drift tube; since the ions pass through focusing ion optics and a quadrupole mass analyzer, space-charge effects may also play a role. It can be inferred from the experiments that ions spend several hundred of microseconds (typically around 500 to 900µs) between the gate and the time of flight mass analyzer. Yet, most interestingly, protein and protein aggregate ions may possess some conformational heterogeneity, which will in turn give rise to broader peaks. Given the low resolution of our ion mobility device ($t/\Delta t$ lying between 3 and 5) it is very likely that several distinct species remain unresolved. Thus, in the case of the $[M+4H]^{4+}$ species (Figure S15a) we may infer the presence of significant conformational variability in addition giving rise to a longtitudal diffusion coefficient that is on average

30-fold greater than calculated from its ionic mobility and equation S4. Another case of interest is the $[2M+7H]^{7+}$ ion. Measured ATDs for this species display a peak with a distinct ‘shoulder’ and a long tail that extends to high drift times. We may label these populations as d7a, d7b and d7c. Treating all three populations as distinct static conformations will yield the cross sections listed in Table ST1. Nevertheless, fitting the data with a linear combination of equation S2 one may observe an interesting phenomenon: whereas D_L for d7a and d7b are overestimated by a similar factor of 17, were the Nernst-Einstein-Townsend relation to hold, the same quantity for d7c is overestimated by a factor of 60!

Table ST2 | Ratios of fitted to expected longtitudal diffusion coefficients for different populations detected in the arrival time spectrum of $[2M+7H]^{7+}$.

Species	Fitted D_L / Expected D_L
d7a	16.8
d7b	16.7
d7c	59.8

Such discrepancy in the peak width of different species in the same ATD point to the fact that d7c differs from the two early-arriving species. The interpretation we suggest is as follows. Whereas d7a and d7b can be viewed as distinct ill-resolved conformational ensembles with minimal interconversion during drift, d7c results from the collapse of a more extended species, which may be either dimeric of originate from an undetected higher order aggregate; it is even possible that d7b is the product of this process. Since ions are stored up to 200x the pusher pulse at the hexapole region preceding the drift tube, this species must be preserved during the ESI process but thermalisation that occurs from the injection of the ions in the drift region must provide the energy required to trigger the change. This result is corroborated by the fact that low collision energies are required to trigger dissociation of the aggregates by CID. A competing interpretation would be the presence of different unresolved conformations giving rise to an apparent tail in the ATD; yet we favour a dynamic picture for the multimeric ions rather insisting on the static model by *arbitrarily* adding more unresolved species.

In short, observed ATDs are much broader than initially expected and this broadening may hint to the dynamic behaviour of the observed ions in the IM mass spectrometer.

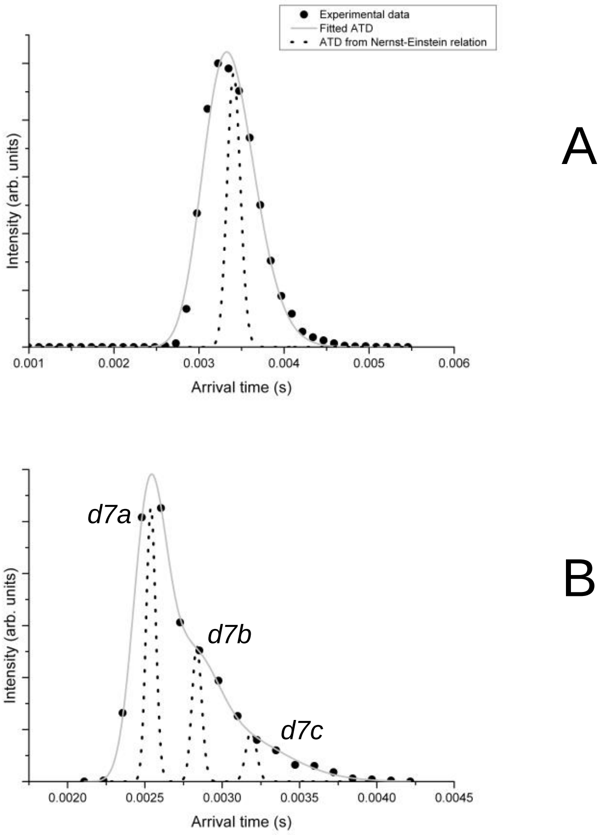

Figure S15 | Arrival time distributions for A) $[M+4H]^{4+}$ and B) $[2M+7H]^{7+}$ ions. Black dots: experimental arrival time spectra; grey curves: ATDs fitted with equation S1; black dotted curves: ATDs calculated with the same expression but by forcing D_L to comply with equation S3. Clearly experimental ATDs are broader than expected from the aforementioned equation. A and B are *not* in scale.

3.2 Source Conditions

All instruments utilised n-ESI sources. The n-ESI source enables the production of ions by charging solutions via the insertion of a thin platinum wire into capillary tips. This produces a Taylor cone plume of ionised droplets which are guided into the mass spectrometer down a voltage gradient. n-ESI capillaries were prepared from glass capillaries (World Precision Instruments, Sarasota, USA) using a micropipette puller (Fleming/Brown P-97 Sutter Instruments, Novato, USA).

For QTOF instruments (QTOF 2 and QTOF Ultima, (Waters, UK)) used an elevated source pressure was used to reduce fragmentation of larger oligomeric species [5]. The source voltages were kept as low as possible to prevent fragmentation of oligomers, whilst preserving sufficient signal intensity. QTOFs were calibrated with NaI and the data was processed using Mass Lynx Software 4.1 (Micromass UK).

3.3 Thioflavin T binding assay

1.5mg/ml insulin samples were freshly prepared as stated above (3.1), filtered (using a 0.2µm filter) and thioflavin T (Th T) was added to a final concentration of 20µM. The

change in Th T fluorescence (excitation wavelength: 440nm, emission wavelength: 485nm) was monitored in a BMG Fluorostar optima plate reader, using clear polystyrene 96-well plates coated with a PEG-like polymer (Corning 96-Well Nonbinding Surface microplates). The plate was sealed and incubated at 60°C, the fluorescence emission being recorded at 10-minute intervals. Thirty 100µL aliquots were recorded on the same microplate (the average of all wells is shown in Figure 1 C main text).

3.4 Transmission Electron Microscopy

Samples were stained for TEM took place in the following fashion, removing any excess liquid after each step with a wedge of filter paper. 3µL of the sample of interest were deposited onto a fomvar-coated copper grid (TAAB) and allowed to rest for 5 minutes. The grids were then washed with a droplet (ca. 10µL) of distilled water and stained for 30-40seconds with 4 microlitres of 1% (m/v) uranyl acetate, before being allowed to dry. TEM images were collected using a Philips CM 120 BioTwin transmission electron microscope.

4. Simulation Methodology

4.1 Molecular Modelling

Multimeric order	monomer	dimer	trimer	tetramer	pentamer	hexamer
	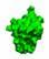	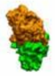	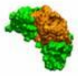	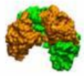	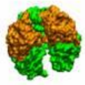	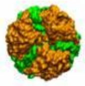
CCS (Å^2)	815.89	1318.6	1729.5	2119.1	2428.7	2586.7

Table ST3 | CCS values for the multimeric species from monomer to hexamer. A structure surface representation for all the species is given: the monomeric units closer to the reader are depicted in orange, whilst the further away units are depicted in green.

4.2 Monomeric species $[M+3H]^{3+}$ and $[M+4H]^{4+}$

Since for a 51 residue protein, reproducing the aqueous medium through an explicit solvent model would make unfolding and folding processes intractable from a computational time point of view, the solvent was represented with a continuum solvation method, termed “OBC” [6,7]. The temperature control at 70°C was effectuated using the Langevin algorithm, with a collision frequency equal to 1.0 ps^{-1}. All the bonds involving hydrogen atoms were constrained at their equilibrium value using the SHAKE algorithm [8], allowing the utilisation of a 2 fs time step.

Discarding the first 2ns (that is 1,000 structures) of the runs, with the remaining 75,000 ones a cluster analysis was performed [9]. Specifically the bottom-up *average-linkage*

algorithm derived 10 clusters of conformations and implemented a sieve of 25. To construct the similarity matrix of distances the backbone RMSD between the pairs of structures was measured. This procedure resulted in 10 conformational families.

4.3 Correlation between CCS and R_g

Through a simulated annealing dynamics loop, 500 structures of the $[M+3H]^{3+}$ species were stored. The implemented loop scheme is reported below:

DO i = 1, 500

heating from 0 K to 800 K in 6.4 ps
dynamics at 800 K for 60 ps
gradual exponential stepwise cooling from 800 K to 0 K, 2 ps per step
minimisation

END DO

The implicit solvent model adopted is the same as above and the time step utilised was 0.002 ps. The runs were conducted with NAMD [10] software.

For the 500 obtained structures, R_g and CCS were calculated. The related scatter plot is given in Figure S16 together with the line of the linear regression (which has a correlation coefficient equal to 0.83).

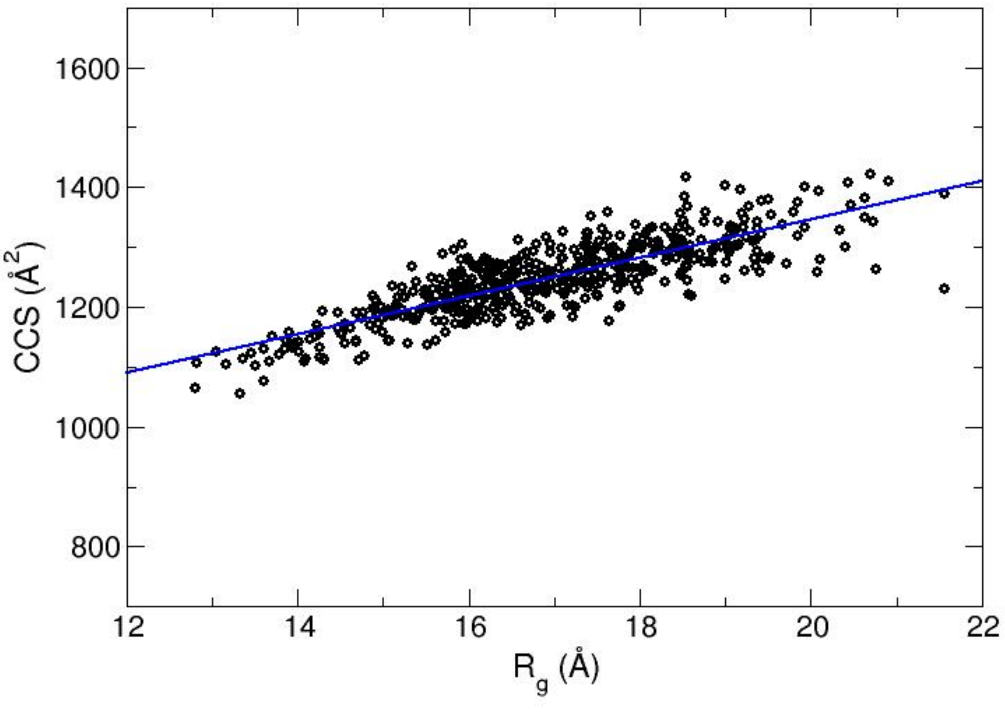

Figure S16 | R_g vs CCS for the $[M+3H]^{3+}$ species. The values were derived from a simulated annealing procedure in implicit solvent.

To reproduce the larger values of the experimental CCS the conformation with the highest R_g value for both +3 and +4 species and were docked, in particular the one at ~33 ns for the +3 species and the one at ~106 ns for the +4 species, applying precisely the same procedure described in the main text for the docking of the most compact monomers. The mean value is equal to 1874.3 Å^2.

4.4 Contact interface and stability of dimers derived from docking

To analyse the interacting residues at the interface between assembled monomeric units, the distribution of the α-carbon pairwise (CA-CA) distances over the 1000 structures derived from the docking procedure have been calculated. The notation $m_i^{(j)}$ was assigned to the monomeric units, where i = I, II, . . . , X characterises a representative structure of one among the ten clusters (only the first two most populated clusters have been considered in this case ie. i = I and II) and j = 3 and 4 stands for the considered net charge. The cut off for the CA-CA distance to be included was set equal to 10 Å.

Essentially the representative structure of the most populated cluster was assembled with itself and with the representative one of the second most populated, obtaining the following families of 1000 dimers each: $m_I^{(3)}m_I^{(3)}$, $m_I^{(3)}m_{II}^{(3)}$, $m_I^{(4)}m_I^{(3)}$, $m_I^{(4)}m_{II}^{(3)}$, $m_I^{(4)}m_I^{(4)}$, $m_I^{(4)}m_{II}^{(4)}$.

A test for checking the interaction energy between the monomers that form dimers is the Molecular Mechanics Poisson Boltzmann Surface Area (MM-PBSA) calculation implemented in Amber software package [11]. Particularly, through this approach, the binding energy of two monomeric structures that form a dimer can be calculated and therefore overcome the “static” picture resulting from the docking, by including dynamical features to the derived dimers.

Even though the energy evaluation is overestimated in absolute value, as the entropic contribution is neglected, this approach gives a sufficiently good relative trend between similar systems [12], like the selected dimers in this work. To perform MM-PBSA calculations the dimers were immersed in a TIP3P [13] water molecules box of approximated dimensions 80 x 70 x 70 Å^3 (varying in according to the dimer under investigation) and containing between ~ 9500 and ~ 11500 water molecules. The systems +6, +7 and +8 charge was neutralized with 6, 7 and 8 chloride ions respectively. After equilibrating temperature (70°C) and density at 1 atm of pressure, a production simulation up to 25 ns in NPT ensemble was run, with temperature collision frequency of 2.0 ps^{-1} and a pressure relaxation time of 2.0 ps. All the bonds involving hydrogen atoms were constrained at their equilibrium values, so that a time step of 2.0 fs could be used and, to derive the electrostatic interactions, particle mesh Ewald (PME) method [14] was implemented, using a radial cut off equal to 8.0A.

A dimer is selected from each $m_i^{(j)}m_k^{(l)}$ family which is representative of the most frequent monomer monomer interface (see Figure S17 below) and is the lowest in energy.

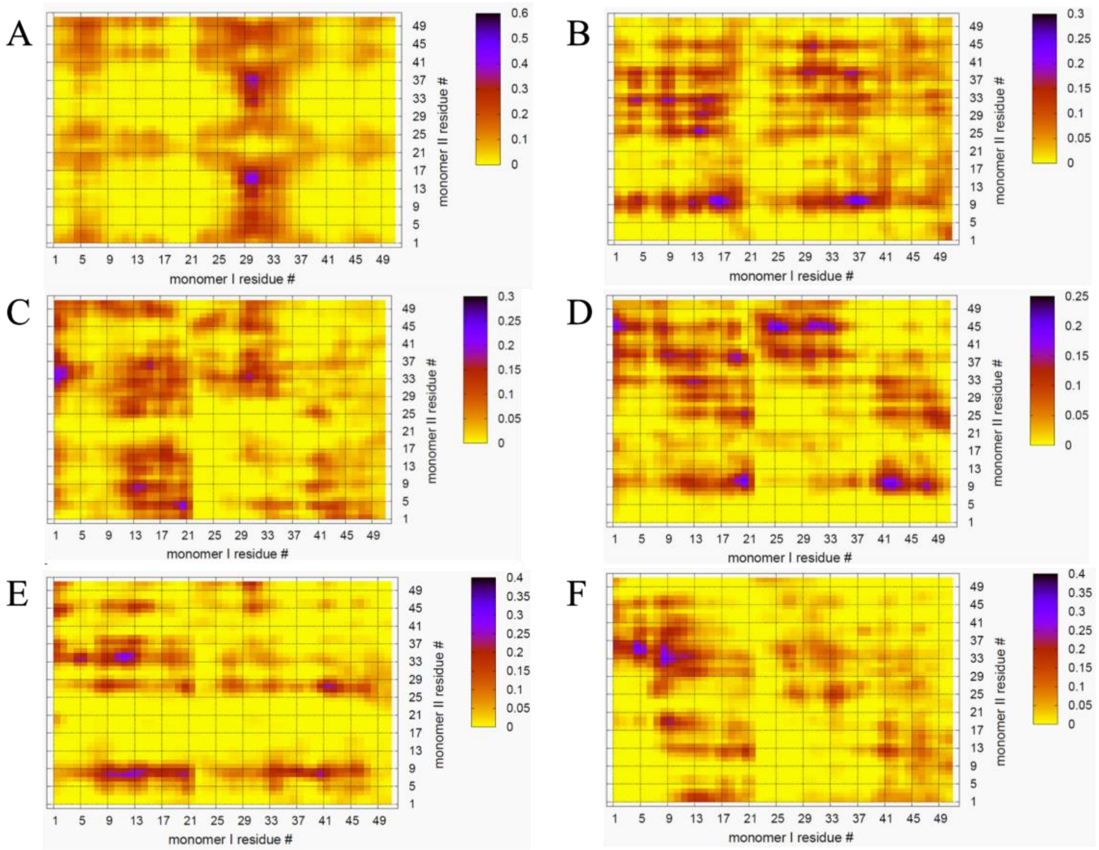

Figure S17 | xy projection of the distributions of the α-carbon pairwise distances. The two upper, middle and lower figures are related to the $[2M+6H]^{6+}$, $[2M+7H]^{7+}$ and $[2M+8H]^{8+}$ species respectively. In particular the figures A, B, C, D, E and F refer to families of docked monomers named $m_I^{(3)}m_I^{(3)}$, $m_I^{(3)}m_{II}^{(3)}$, $m_I^{(4)}m_I^{(3)}$, $m_I^{(4)}m_{II}^{(3)}$, $m_I^{(4)}m_I^{(4)}$, *and* $m_I^{(4)}$ $m_{II}^{(4)}$ respectively.

4.4.1 Breakdown of the contributions to the binding energy of representative dimers

	A	B	C	D	E	F
Hydrophobic (kcal/mol)	-6.8	-8.7	-7.4	-6.2	-10.4	-4.9
Hydrophilic (kcal/mol)	103.6	13.0	21.6	-27.7	49.9	-66.9

Table ST4 | CCS Hydrophobic and hydrophilic contributions to the solvation energy of the dimers representative structures. The hydrophobic and hydrophilic contributions are derived from surface area (SA) and Poisson-Boltzmann (PB) approach respectively, along the MM-PBSA procedure. Letters A, B, C, D, E and F refer to the representative dimers of the families $m_I^{(3)}m_I^{(3)}$, $m_I^{(3)}m_{II}^{(3)}$, $m_I^{(4)}m_I^{(3)}$, $m_I^{(4)}m_{II}^{(3)}$, $m_I^{(4)}m_I^{(4)}$, *and* $m_I^{(4)}$ $m_{II}^{(4)}$ respectively.

4.4.2 CCSs of dimer structures from dynamics in water solvent

During the dynamics in water, 25 dimer structures were stored for each family. Water molecules were stripped out and minimisation *in vacuo* was performed. The calculated CCSs are displayed in Figure S18 below.

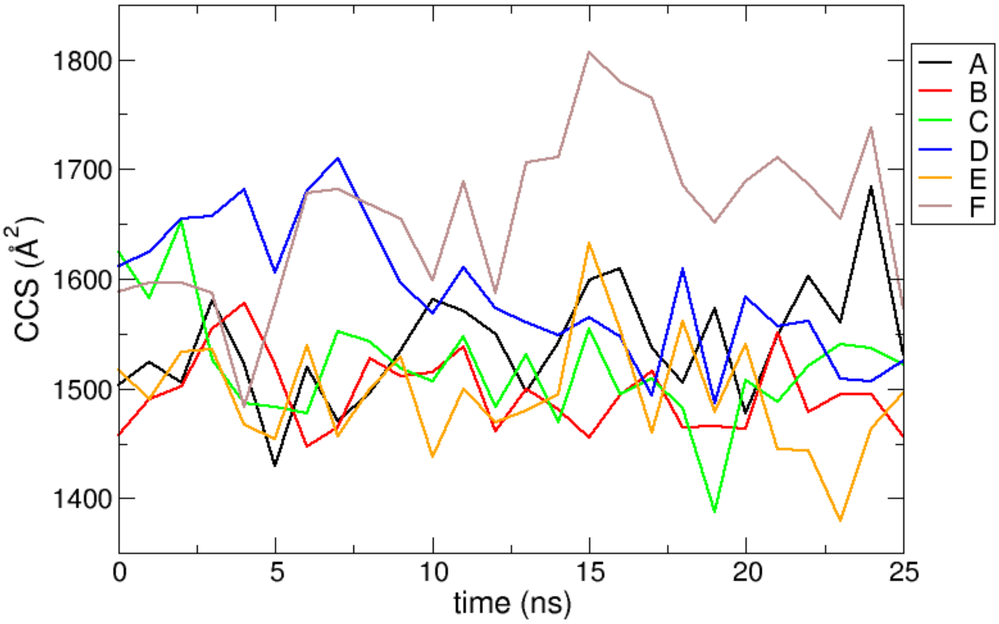

Figure S18 | Time series for CCS versus simulation time for the dimers. The lines marked with letters A, B, C, D, E and F in the legend box refer to the selected dimers from the families $m_I^{(3)}m_I^{(3)}$, $m_I^{(3)}m_{II}^{(3)}$, $m_I^{(4)}m_I^{(3)}$, $m_I^{(4)}m_{II}^{(3)}$, $m_I^{(4)}m_I^{(4)}$, *and* $m_I^{(4)}m_{II}^{(4)}$ respectively.

5. References

1 Clemmer, D. E. & Jarrold, M. F. Ion mobility measurements and their applications to clusters and biomolecules. *J. Mass Spectrom.* **32**, 577-592 (1997).

2 Jarrold, M. F. Peptides and proteins in the vapor phase. *Annual Review of Physical Chemistry* **51**, 179-207 (2000).

3 Smith, D. P., Radford, S. E. & Ashcroft, A. E. Elongated oligomers in β_2-microglobulin amyloid assembly revealed by ion mobility spectrometry-mass spectrometry. *Proc. Natl. Acad. Sci. U. S. A.* **107**, 6794-6798, doi:10.1073/pnas.0913046107 (2010).

4 Moseley, J. T., Gatland, I. R., Martin, D. W. & McDaniel, E. W. Measurement of Transport Properties of Ions in Gases - Results for K+ Ions in N2. *Physical Review* **178**, 234-&, doi:10.1103/PhysRev.178.234 (1969).

5 Sobott, F., Hernandez, H., McCammon, M. G., Tito, M. A. & Robinson, C. V. A tandem mass spectrometer for improved transmission and analysis of large macromolecular assemblies. *Anal. Chem.* **74**, 1402-1407, doi:10.1021/ac0110552 (2002).
6 Onufriev, A., Bashford, D. & Case, D. A. Exploring protein native states and large-scale conformational changes with a modified generalized born model. *Proteins-Structure Function and Bioinformatics* **55**, 383-394, doi:10.1002/prot.20033 (2004).
7 Feig, M. *et al.* Performance comparison of generalized born and Poisson methods in the calculation of electrostatic solvation energies for protein structures. *Journal of Computational Chemistry* **25**, 265-284, doi:10.1002/jcc.10378 (2004).
8 Tildesley, M. P. A. a. D. J. *Computer simulation of liquids*. (Clarendon Press, 1986).
9 Shao, J. Y., Tanner, S. W., Thompson, N. & Cheatham, T. E. Clustering molecular dynamics trajectories: 1. Characterizing the performance of different clustering algorithms. *Journal of Chemical Theory and Computation* **3**, 2312-2334, doi:10.1021/ct700119m (2007).
10 Phillips, J. C. *et al.* Scalable molecular dynamics with NAMD. *Journal of Computational Chemistry* **26**, 1781-1802, doi:10.1002/jcc.20289 (2005).
11 Kollman, P. A. *et al.* Calculating Structures and Free Energies of Complex Molecules: Combining Molecular Mechanics and Continuum Models. *Accounts Chem. Res.* **33**, 889-897, doi:10.1021/ar000033j (2000).
12 Gilson, M. K. & Zhou, H. X. Calculation of protein-ligand binding affinities. *Annual Review of Biophysics and Biomolecular Structure* **36**, 21-42, doi:10.1146/annurev.biophys.36.040306.132550 (2007).
13 Jorgensen, W. L., Chandrasekhar, J., Madura, J. D., Impey, R. W. & Klein, M. L. Comparison of Simple Potential Functions for Simulating Liquid Water. *J. Chem. Phys.* **79**, 926-935 (1983).
14 Darden, T., York, D. & Pedersen, L. Particle Mesh Ewald - an n.log(n) Method for Ewald Sums in Large Systems. *J. Chem. Phys.* **98**, 10089-10092 (1993).
